# Mean-Field Models for EEG/MEG: From Oscillations to Waves

**DOI:** 10.1007/s10548-021-00842-4

**Published:** 2021-05-15

**Authors:** Áine Byrne, James Ross, Rachel Nicks, Stephen Coombes

**Affiliations:** 1 School of Mathematics and Statistics, Science Centre, University College Dublin, South Belfield, Dublin 4, Ireland; 2 School of Mathematical Sciences, Centre for Mathematical Medicine and Biology, University of Nottingham, Nottingham, NG7 2RD UK

**Keywords:** Neural mass, Neural field, Brain rhythms, Synchrony, Waves, Synaptic coupling, Gap-junction coupling

## Abstract

Neural mass models have been used since the 1970s to model the coarse-grained activity of large populations of neurons. They have proven especially fruitful for understanding brain rhythms. However, although motivated by neurobiological considerations they are phenomenological in nature, and cannot hope to recreate some of the rich repertoire of responses seen in real neuronal tissue. Here we consider a simple spiking neuron network model that has recently been shown to admit an exact mean-field description for both synaptic and gap-junction interactions. The mean-field model takes a similar form to a standard neural mass model, with an additional dynamical equation to describe the evolution of within-population synchrony. As well as reviewing the origins of this *next generation* mass model we discuss its extension to describe an idealised spatially extended planar cortex. To emphasise the usefulness of this model for EEG/MEG modelling we show how it can be used to uncover the role of local gap-junction coupling in shaping large scale synaptic waves.

**Supplementary Information:**

The online version contains supplementary material available at 10.1007/s10548-021-00842-4.

## Introduction

The use of mathematics has many historical successes, especially in the fields of physics and engineering, where mathematical concepts have been put to good use to address challenges far beyond the context in which they were originally developed. Physicists in particular are well aware of the “The Unreasonable Effectiveness of Mathematics in the Natural Sciences” (Wigner 1960). One recent breakthrough in the field of large-scale brain modelling has come about because of advances in obtaining exact mean-field reductions of certain classes of coupled oscillator networks via the so-called Ott–Antonsen (OA) ansatz (Ott and Antonsen 2008). This is especially important because the mathematical step from microscopic to macroscopic dynamics has proved elusive in all but a few special cases. Indeed, many of the current models used to describe coarse-grained neural activity, such as the Wilson-Cowan (Wilson and Cowan 1972), Jansen-Rit (Jansen and Rit 1995), or Liley (Liley et al. 2002) model are phenomenological in nature. Nonetheless they have been used extensively to study and explore the potential mechanisms that coordinate brain rhythms underlying cognitive processing and large scale neuronal communication (Fries 2005). For example, such *neural mass* models have recently been used to understand cross-frequency coupling between brain areas (Jedynak et al. 2015), understand how patterns of functional connectivity may arise in brain imaging studies (Forrester et al. 2020), and are a key ingredient of the Virtual Brain project that aims to deliver the first open simulation of the human brain based on individual large-scale connectivity (Sanz-Leon et al. 2015). Moreover they have been used to uncover how hyper- and hypo-synchrony of neuronal network firing may underpin brain dysfunction including epilepsy (Wendling et al. 2016).

Making use of the OA reduction Luke and colleagues (Luke et al. 2013; So et al. 2014) were able to obtain exact asymptotic dynamics for networks of pulse-coupled theta-neurons (Ermentrout and Kopell 1986). Although the theta-neuron model is simplistic, it is able to capture some of the essential features of cortical firing pattern, such as low firing rates. As such, this mean-field reduction is a candidate for a new type of cortical neural mass model that makes a stronger connection to biological reality than the phenomenological models mentioned above. The theta-neuron is formally equivalent to the quadratic integrate-and-fire (QIF) model (Latham et al. 2000), a mainstay of many studies in computational neuroscience, e.g. Dipoppa and Gutkin (2013). Interestingly an alternative to the OA approach has been developed by Montbrió et al. (2015) that allows for an equivalent reduction of networks of pulse-coupled QIF neurons, and establishes an interesting duality between the two approaches. In the OA approach the complex Kuramoto order parameter is a fundamental macroscopic variable and the population firing rate is a function of the degree of dynamically evolving within-population synchrony. Alternatively in the approach of Montbrió et al. average voltage and firing rate couple dynamically to describe emergent population behaviour. Given that both approaches describe the same overall system *exactly* (at least in the thermodynamic limit of an infinite number of neurons) there must be an equivalence between the two macroscopic descriptions. Montbrió et al. have further shown that this relationship takes the form of a conformal map between the two physical perspectives. This correspondence is very useful when dealing with different types of neuroimaging modality. For example, when looking at power spectrograms from electro- or magneto-encephalograms (EEG/MEG), it is useful to contemplate the Kuramoto order parameter since changes in coherence (synchrony) of spike trains are likely to manifest as changes in power. On the other hand the local field potential recorded by an extracellular electrode may more accurately reflect the average population voltage. A model with a perspective on both, simply by a mathematical change of viewpoint, is not only useful for describing experimental data, it may also help the brain imaging community develop new approaches that can exploit a non-intuitive link between seemingly disparate macroscopic variables. Importantly, for this to be relevant to the real world some further features of neurobiology need to be incorporated, as purely pulsatile coupling is not expected to capture all of the rich behaviour seen in brain oscillations and waves. In particular synaptic processing and gap-junction coupling at the level of localised populations of neurons, and axonal delays at the larger tissue scale are all well known to make a major contribution to brain rhythms, both temporal and spatio-temporal (Nunez and Srinivasan 2005; Buzsáki 2011). Fortunately, these biological extensions, that generalise the initial theta-neuron and QIF network models with pulsatile coupling, are natural and easily accommodated. Work in this area has already progressed, e.g. with theoretical work by Laing (2015) and Pietras et al. (2019) on how to treat gap junctions, and by Coombes and Byrne (2019) on the inclusion of realistic synaptic currents (governed by reversal potentials and dynamic conductance changes). Recent work by Byrne et al. (2020) has also considered the inclusion of finite action potential speeds. In this paper we consider a synthesis of modelling work to date on developing a new class of mean-field models fit for use in complementing neuroimaging studies, and present some new results emphasising the important role of local gap-junction coupling in shaping brain rhythms and waves.

Even without the inclusion of gap junctions a first major success of this so-called *next generation* neural mass and field modelling approach has been in explaining the phenomenon of beta-rebound. Here a sharp decrease in neural oscillatory power in the 15 Hz EEG/MEG beta band is observed during movement followed by an increase above baseline on movement cessation. Standard neural mass models cannot readily reproduce this phenomenon, as they cannot track changes of synchrony within a population. On the other hand the next-generation models treat population coherence as fundamental, and are able to track and describe changes in synchrony in a way consistent with movement-related beta decrease, followed by an increase above baseline upon movement termination (post-movement beta rebound) (Byrne et al. 2017). Moreover, these models are capable of explaining the abnormal beta-rebound seen in patients with schizophrenia (Byrne et al. 2019). Beta decrease and rebound are special cases of event related synchrony/de-synchrony (ERS/ERD), as measured by changes in power at given frequencies in EEG/MEG recordings (Pfurtscheller and da Silva 1999), and as such this class of model clearly has wider applicability than standard neural mass models that cannot describe ERD/ERS because their level of coarse-graining does not allow one to interrogate the degree of within-population synchrony. By merging this new dynamical model of neural tissue with anatomical connectome data it has also been possible to gain a perspective on whole brain dynamics, and preliminary work in Byrne et al. (2020) has given insight into how patterns of resting state functional-connectivity can emerge and how they might be disrupted by transcranial magnetic stimulation.

Despite the success of the next generation models that include synaptic processing it is well to recognise the importance of direct electrical communication between neurons that can arise via gap junctions. Without the need for receptors to recognise chemical messengers gap junctions are much faster than chemical synapses at relaying signals. The communication delay for a chemical synapse is typically in the range 1–100 ms, while that for an electrical synapse may be only about 0.2 ms. Gap junctions have long been thought to be involved in the synchronisation of neurons (Alvarez et al. 2002; Bennet and Zukin 2004) and are believed to contribute to both normal (Hormuzdi et al. 2004) and abnormal physiological brain rhythms, including epilepsy (Velazquez and Carlen 2000; Martinet et al. 2017).

In the “Neural Mass Model” section﻿ we introduce the mathematical description for the microscopic spiking cell dynamics as a network of QIF neurons with both synaptic and gap-junction coupling. We present the corresponding mean-field ordinary differential equation model with a focus on the bifurcation properties of the model under variation of key parameters, including the level of population excitability and the strength of gap-junction coupling. A simple cortical model built from two sub-populations, one excitatory and the other inhibitory, is shown to produce robust oscillations via a Hopf bifurcation. The derivation of the macroscopic equations of motion is deferred to a technical appendix. This new class of neural mass model is used as a building block in “Neural Field Model” section to construct a continuum model of cortical tissue in the form of an integro-differential neural field model. Here, long-range connections are mediated by action potentials giving rise to space-dependent axonal delays. For computational ease we reformulate the neural field as a brain-wave partial differential equation, and pose it on idealised one- and two-dimensional spatial domains. A Turing analysis is performed to determine the onset of instabilities that lead to novel patterned states, including bulk oscillations and periodic travelling waves. These theoretical predictions, again with details deferred to a technical appendix, are confirmed against direct numerical simulations. Moreover, beyond bifurcation we show that the tissue model can support rich rotating structures, as well as localised states with dynamic cores. Finally, in the “Discussion” section we outline further applications and extensions of the work presented in this paper.

## Neural Mass Model

Here we describe a new class of neural mass model that can be derived from a network of spiking neurons. The microscopic dynamics of choice is the QIF neuron model, which is able to replicate many of the properties of cortical cells, including a low firing rate. In contrast to the perhaps more well studied linear or leaky IF model it is also able to represent the shape of an action potential. This is important when considering electrical synapses, whereby neurons directly “feel” the shape of action potentials from other neurons to which they are connected. An electrical synapse is an electrically conductive link between two adjacent nerve cells that is formed at a fine gap between the pre- and post-synaptic cells known as a gap junction and permits a direct electrical connection between them. They are now known to be ubiquitous throughout the human brain, being found in the neocortex (Galarreta and Hestrin 1999), hippocampus (Fukuda and Kosaka 2000), the inferior olivary nucleus in the brain stem (Sotelo et al. 1974), the spinal cord (Rash et al. 1996), the thalamus (Hughes and Crunelli 2007) and have recently been shown to form axo-axonic connections between *excitatory* cells in the hippocampus (on mossy fibers) (Hamzei-Sichani et al. 2007). It is common to view the gap junction as nothing more than a channel that conducts current according to a simple ohmic model. For two neurons with voltages $v_i$ and $v_j$ the current flowing into cell *i* from cell *j* is proportional to $v_j-v_i$. This gives rise to a *state-dependent* interaction. In contrast, chemical synaptic currents are better modelled with *event-driven* interactions. If we denote the *m*th firing time of neuron *j* by $T_j^m$ then the current received by neuron *i* if connected to neuron *j* would be proportional to $\sum _{m \in \mathbb {Z}} s(t- T_j^m)$, where *s* is a temporal shape that describes the typical rise and fall of a post synaptic response. This is often taken to be the Green’s function of a linear differential operator *Q*, so that $Q s =\delta$ where $\delta$ is a delta-Dirac spike. Throughout the rest of this paper we shall take $s(t) = \alpha ^2 t \exp (-\alpha t) H(t)$, where *H* is a Heaviside step function. In this case the operator *Q* is second order in time and given by

1 $$\begin{aligned} Q = \left( 1 + \frac{1}{\alpha } \frac{\mathrm{d}}{\mathrm{d}t}\right) ^ 2, \end{aligned}$$

where $\alpha ^{-1}$ is the time-to-peak of the synapse.

We are now in a position to consider a heterogeneous network of *N* quadratic integrate-and-fire neurons with voltage $v_i$ and both gap-junction and synaptic coupling:

2 $$\begin{aligned} \tau \dot{v}_i = \eta _i+v_i^2 + \frac{\kappa _v}{N} \sum _{j=1}^N (v_j - v_i) + \frac{\kappa _s}{N} \sum _{j=1}^N \sum _{m \in \mathbb {Z}} s(t- T_j^m), \end{aligned}$$

**Fig. 1:**
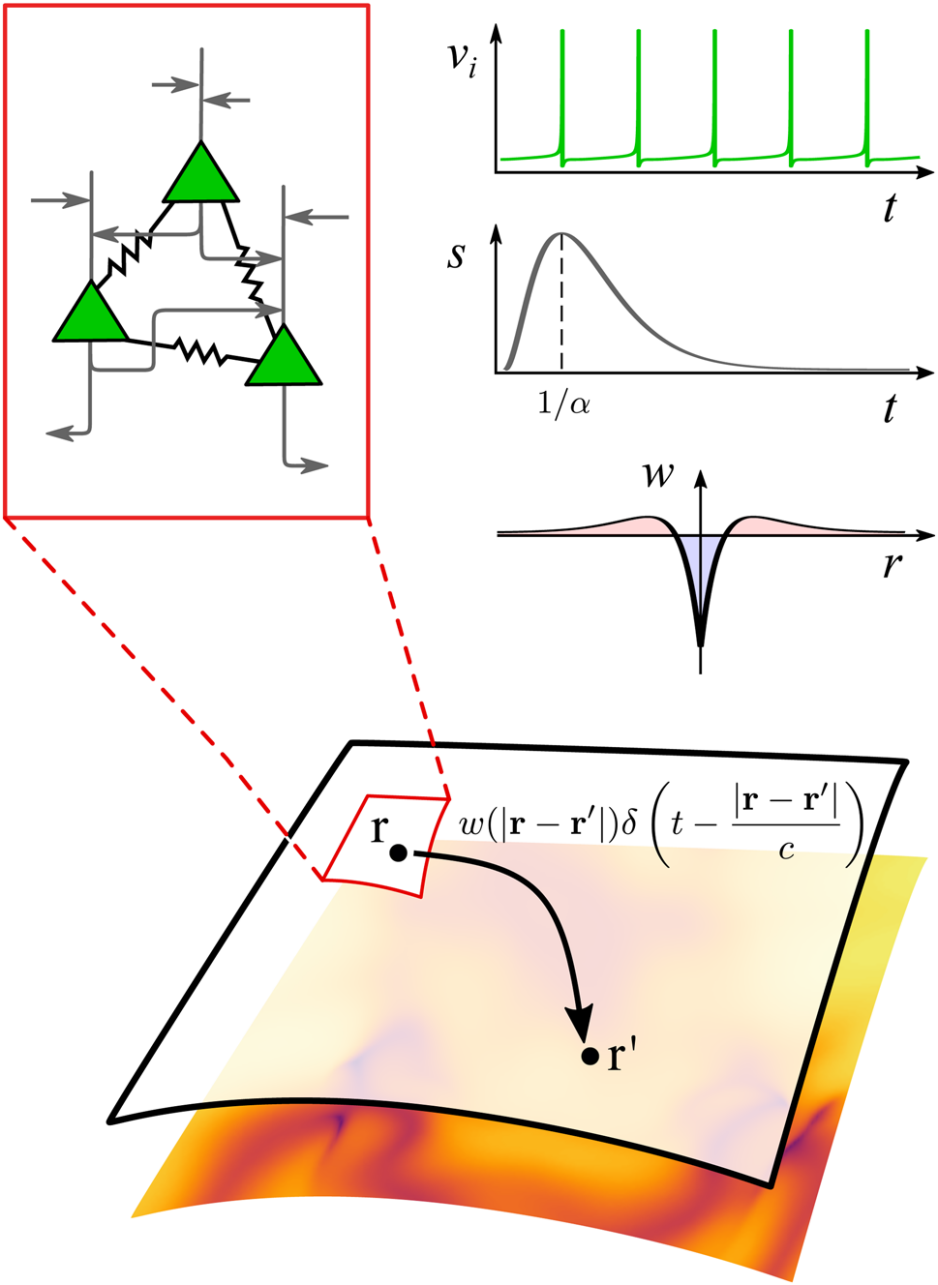
Model schematic. At each point in a two-dimensional spatial continuum there resides a density of QIF neurons whose mean-field dynamics are described by the triple (*R*, *V*, *U*), where *R* represents population firing rate, *V* the average membrane potential, and *U* the synaptic activity. The non-local interactions are described by a kernel *w*, taken to be a function of the distance between two points. The space-dependent delays arising from signal propagation along axonal fibres are determined in terms of the speed of the action potential *c*

$i=1,\ldots , N$, with $v_\text {r} \le v_i \le v_\text {th}$. Here, firing times are defined implicitly by $v_j(T_j^m)=v_\text {th}$. The network nodes are subject to reset: $v_i \rightarrow v_\text {r}$ at times $T_i^m$. The parameter $\tau$ is the membrane time constant. The strengths of gap-junction and synaptic coupling are $\kappa _v$ and $\kappa _s$ respectively. The background inputs $\eta _i$ are random variables drawn from a Lorentzian distribution with median $\eta _0$ and half width $\gamma$. The value of $\eta _0$ can be thought of as setting the level of excitability, and $\gamma$ as the degree of heterogeneity in the network. The larger $\eta _0$ is, the more neurons would fire if uncoupled, and the larger $\gamma$ is, the more dissimilar the inputs are. A schematic of a QIF network and its reduction to a neural field model is shown in Fig. 1, with details of the neural field formulation described in “Neural Field Model” section.

**Fig. 2:**
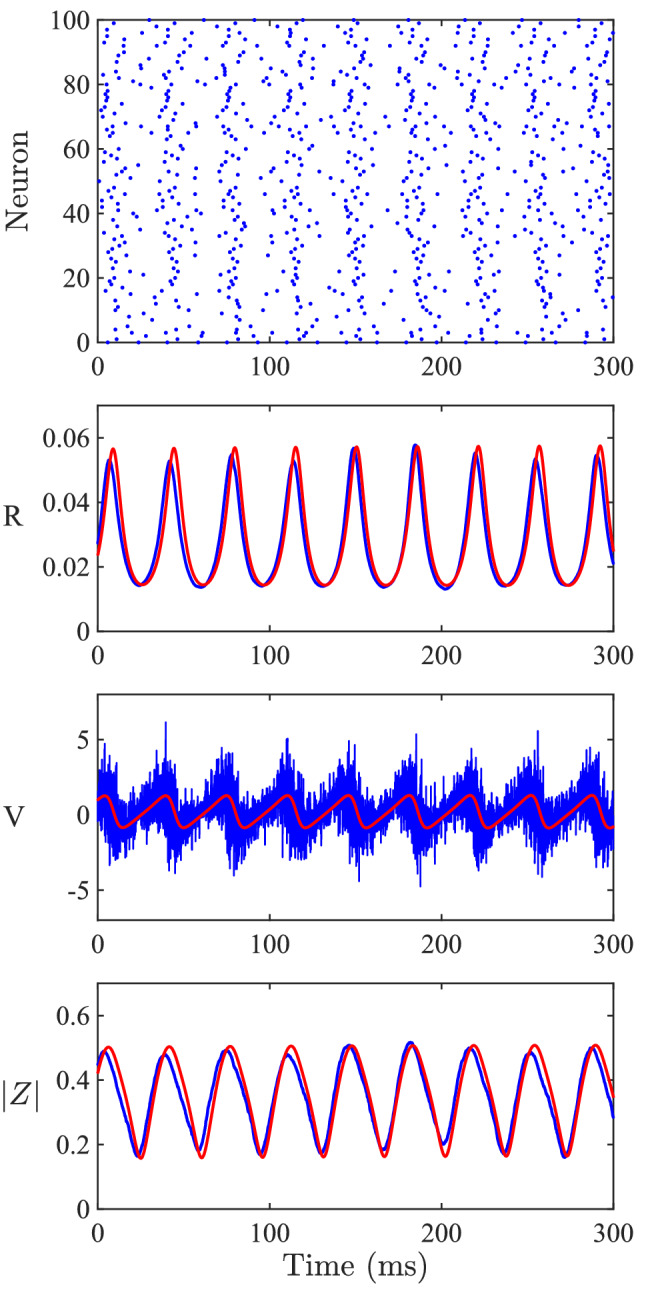
Validity of the mean-field reduction. A comparison of the mean-field dynamics (red) with the corresponding network of spiking neurons (blue). The top panel shows a raster plot for a sample of 100 of the 1000 neurons in the network of synaptically and electrically coupled QIF neurons. Below are comparisons of the mean firing rate *R*, average membrane potential *V* and within population synchrony |*Z*| for the spiking network and mean field model. Parameter values: $\eta _0= 2$, $\kappa _v = 1$, $\kappa _s =1$, $\tau = 16$, $\alpha =0.5$, $\gamma = 0.5$. For the spiking network simulations $v_\text {r}=-1000$ and $v_\text {th}=1000$, while in the mean field limit these are assumed to be $-\infty$ and $\infty$, respectively

The mean-field reduction of (2) can be achieved by using the approach of Montbrió et al. (2015). This is described in detail in Appendix 1, and is valid for globally coupled cells in the thermodynamic limit $N\rightarrow \infty$. The network behaviour can be summarised by the instantaneous mean firing rate *R*(*t*) (the fraction of neurons firing at time *t*), the average membrane potential *V*(*t*) ($=\lim _{N \rightarrow \infty } N^{-1}\sum _{i=1}^N v_i$), and the synaptic activity *U*(*t*). The synaptic activity *U* is driven by mean firing rate according to $Q U = R$, with the mean-field dynamical equations for (*R*, *V*):

3 $$\begin{aligned} \tau \dot{R}&= - \kappa _v R + 2 R V + \frac{\gamma }{\pi \tau } , \end{aligned}$$

4 $$\begin{aligned} \tau \dot{V}&= \eta _0 + V^2- \pi ^2 \tau ^2 R^2 + \kappa _s U . \end{aligned}$$

**Fig. 3:**
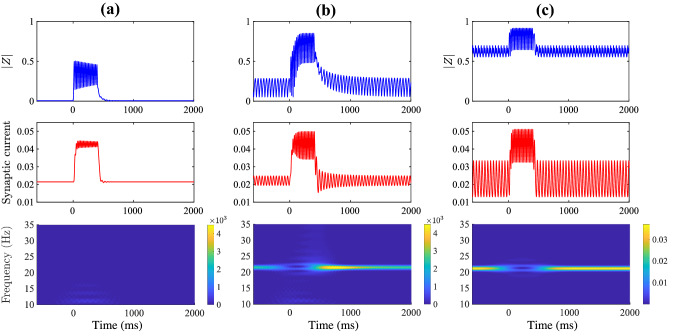
Beta rebound. Time course of the within population synchrony and synaptic current ($\kappa _s U$)) and a time-frequency spectrogram of the synaptic current for different gap-junction coupling strengths when a temporally filtered square pulse of length 400 ms and magnitude 3 μA was applied to the model (3)–(4). **a** Weak gap-junction coupling $\kappa _v=0.5$. **b** Intermediate gap-junction coupling $\kappa _v=1$. **c** Strong gap-junction coupling $\kappa _v=1.5$. Parameter values: $\eta _0= 1$, $\kappa _s =1$, $\tau = 15$, $\alpha =0.1$, $\gamma = 0.5$

Interestingly this (*R*, *V*) perspective on population dynamics can be mapped to one that tracks the degree of within-population synchrony described by the complex Kuramoto order parameter *Z* according to the conformal map (Montbrió et al. 2015):

5 $$\begin{aligned} Z = \frac{1-W^*}{1+W^*} , \qquad W=\pi \tau R +i V, \end{aligned}$$

where $W^*$ is the complex conjugate of *W*. The corresponding dynamics for *Z* is given by equation (23) in Appendix 1. Alternatively, one can evolve the model for (*R*, *V*, *U*) and then obtain results about synchrony |*Z*| by the use of (5).

The mean field model accurately describes the underlying spiking network (Fig. 2). A network of 1000 synaptically and electrically coupled QIF neurons (blue), as described by (2), was simulated and compared to the mean field dynamics (red), as described by (3) and (4). The finite size fluctuations are most apparent for the membrane potential *V*. However, the overall behaviour is similar. As expected, increasing the population size reduces the finite size fluctuations.

A previous instance of this model, without gap-junction coupling and with synaptic reversal potentials, was applied to describe beta-rebound, as seen in real MEG data (Byrne et al. 2017). Beta-rebound is a special case of event-related desynchronisation and synchronisation, whereby power in the beta band decreases at movement initiation and rebounds above baseline after movement termination. For our model, which does not incorporate synaptic reversal potentials, we find that gap-junction coupling is important for beta-rebound. In particular, our results suggest that there is a delicate balance between too little gap-junction coupling and too much gap-junction coupling (Fig. 3). A temporally filtered square pulse of length 400 ms and magnitude 3 μA was applied to the model at $t=0$ ms to mirror a movement. For intermediate values of gap-junction coupling, there is a reduction in beta power at movement onset (0 ms), followed by a sharp increase in power shortly after movement termination (500 ms). The transient increase in beta band power presents with high within population synchrony, confirming the link between rebound and synchronisation. With weak gap-junction coupling, the system does not oscillate, and as such, beta rebound is not possible. With strong gap-junction coupling, the system oscillates, but after movement termination the system returns, almost immediately, to its original behaviour. The population is overly synchronised at steady state, and as such, a transient of high synchrony is not possible.

**Fig. 4:**
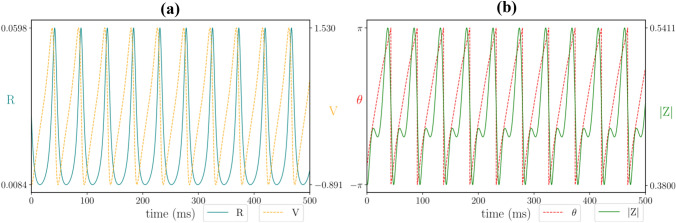
Single population dynamics. **a** Oscillations in the population firing rate *R* (teal) and average membrane voltage *V* (yellow), **b** Corresponding oscillations in the complex Kuramoto order parameter $Z=|Z|\mathrm{e}^{i \theta }$, where |*Z*| reflects the degree of within-population synchrony (green), and $\theta$ a corresponding phase (red). Parameter values: $\eta _0= 1$, $\kappa _v = 1.2$, $\kappa _s =1$, $\tau = 15$, $\alpha =0.5$, $\gamma = 0.5$

### Single Population: Bifurcation Analysis

We first consider a single excitatory population ($\kappa _s>0$). In contrast to a scalar rate model with self-feedback (as an exemplar of a single population model), the next generation model has at least two variables (to describe either synchrony *Z*, or the pair (*R*, *V*)) and thus is of high enough dimension to support oscillations in time (Fig. 4). Examining the profile of these oscillations, we observe that the peaks and troughs of the firing rate *R* and the synchrony |*Z*| roughly coincide. This indicates, rather unsurprisingly, that when a population is highly synchronised the population firing rate will be high.

**Fig. 5:**
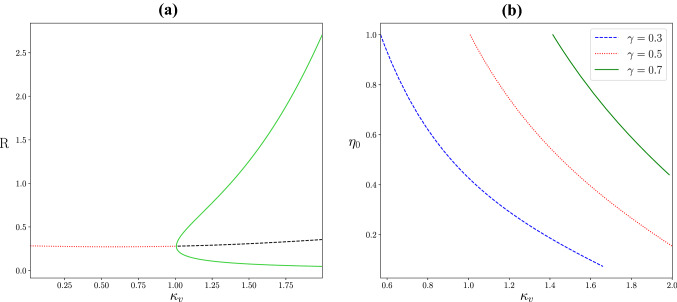
Single population bifurcation diagrams. **a** A Hopf bifurcation is found with an increase in the strength of gap-junction coupling $\kappa _v$, giving rise to limit cycle oscillations. Red (black) lines denote the stable (unstable) fixed point, while the green lines show the minimum and maximum of the oscillation. **b** A two parameter bifurcation diagram in the $(\kappa _v,\eta _0)$-plane tracing the locus of Hopf bifurcations for different values of $\gamma$. Oscillations emerge to the right of each curve. Parameter values: $\eta _0= 1$, $\kappa _s =1$, $\tau = 15$, $\alpha =0.5$, $\gamma = 0.5$

As the strength of gap-junction coupling $\kappa _v$ is decreased the system undergoes a Hopf bifurcation and oscillations disappear (Fig. 5). Note that to the right of the Hopf bifurcation the amplitude and frequency of the oscillations increases with $\kappa _v$. Increasing the level of excitability $\eta _0$ also leads to oscillatory behaviour. A continuation of the Hopf bifurcation in $\kappa _v$ and $\eta _0$ is shown for different values of $\gamma$ (Fig. 5). The system oscillates for parameter values to the right of these curves. Remembering that $\gamma$ sets the level of heterogeneity, we note the window for oscillations gets smaller as the heterogeneity of network is increased.

### Excitatory-Inhibitory Network: Bifurcation Analysis

The single population model can be easily extended to a two population network, consisting of an excitatory and an inhibitory population, labelled by *E* and *I* respectively. Synaptic coupling is present both within and between populations, while gap-junction coupling only exists between neurons in the same population. The augmented system of equations, describing the mean firing rate *R* and the average membrane potential *V* of each population, as well as 4 distinct synaptic variables *U* for each of the synaptic connections, is presented in Appendix 2.

**Fig. 6:**
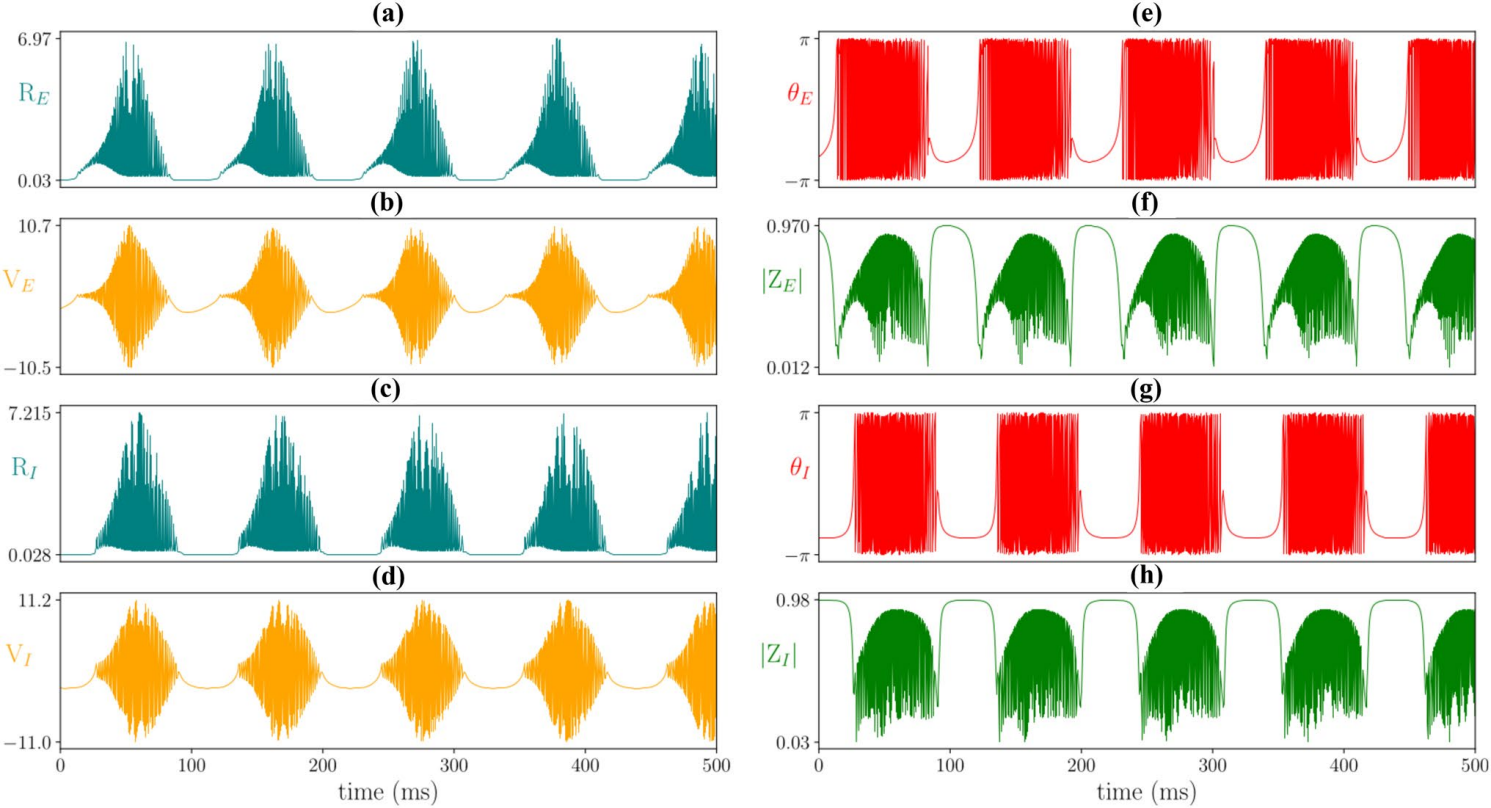
Excitatory-inhibitory network dynamics: Oscillations in **a** the excitatory population firing rate $R_E$ (teal), and **b** in the average membrane potential $V_E$ (yellow). Corresponding oscillations for the inhibitory population, **c** $R_I$ and **d** $V_I$. Kuramoto order parameters for the excitatory population **e** $Z_E=|Z_E|\mathrm{e}^{i \theta _E}$, $|Z_E|$ (green) and **f** $\theta _E$ (red). Corresponding traces for **g** $|Z_I|$ and **h** $\theta _I$ of the inhibitory population. Parameter values: $\eta _0^E= 5$, $\eta _0^I= -3$, $\kappa _v^E =\kappa _v^I= 0.5$, $\kappa _s^{EE} =15$, $\kappa _s^{IE} =25$, $\kappa _s^{EI} =-15$, $\kappa _s^{II} =-15$, $\tau _E = 1, \tau _I = 1$, $\alpha _{EE}=0.2$, $\alpha _{IE}=0.1$, $\alpha _{EI}=0.07$, $\alpha _{II}=0.06$, $\gamma _E = \gamma _I = 0.5$

The excitatory-inhibitory network possesses a rich repertoire of dynamics. For example, it is possible to generate bursts of high frequency and high amplitude activity at a slow burst rate (Fig. 6). This pattern of activity is typical in epileptic seizures, e.g. (Krishnan et al. 2013). Decreasing the gap-junction coupling strengths $\kappa _v^E$ and $\kappa _v^I$ results in smoother lower amplitude oscillations, more in line with healthy brain oscillations. We note that $\kappa _v^E$ and $\kappa _v^I$ are not the only parameters that can change the profile of the oscillations; reducing $\eta _0^E$ (the median background drive to the excitatory population) can also eradicate the seizure-like oscillations.

**Fig. 7:**
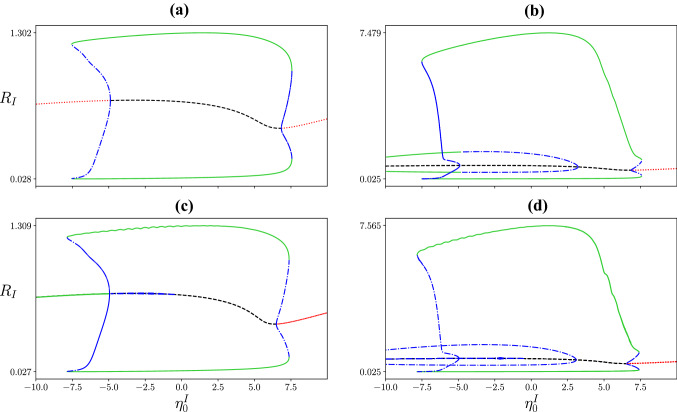
Two population bifurcation diagrams: Continuations in the median background drive to the inhibitory population $\eta _0^I$ for different combinations of gap-junction coupling strengths $\kappa _v^E$ and $\kappa _v^I$. Red (black) lines denote the stable (unstable) fixed point, while the green (blue) lines show the minimum and maximum of the stable (unstable) oscillation **a** No gap-junction coupling, $\kappa _v^E = 0$, $\kappa _v^I = 0$, **b** Gap junctions in the inhibitory population only, $\kappa _v^E = 0$, $\kappa _v^I = 0.5$, **c** Gap junctions in the excitatory population only, $\kappa _v^E = 0.5$, $\kappa _v^I = 0$, **d** Gap junction coupling in both populations, $\kappa _v^E = 0.5$, $\kappa _v^I = 0.5$. Other parameters: $\eta _0^E= 5$, $\kappa _s^{EE} =15$, $\kappa _s^{IE} =25$, $\kappa _s^{EI} =-15$, $\kappa _s^{II} =-15$, $\tau _E = 1,\tau _I = 1$, $\alpha _{EE}=0.2$, $\alpha _{IE}=0.1$, $\alpha _{EI}=0.07$, $\alpha _{II}=0.06$, $\gamma _E = \gamma _I = 0.5$

Next we examine the bifurcation structure of the excitatory-inhibitory network for different combinations of gap-junction coupling strengths $\kappa _v^E$ and $\kappa _v^I$ (Fig. 7). With no gap-junction coupling in either population ((a) $\kappa _v^E=\kappa _v^I=0$), intermediate values of the median background drive to the inhibitory population $\eta _0^I$ result in oscillatory behaviour. Switching on the gap-junction coupling in the inhibitory population only ((b) $\kappa _v^E=0$, $\kappa _v^I=0.5$), the amplitude of oscillation increases significantly for this branch of oscillatory solutions. An additional branch of oscillatory solutions emerges for low $\eta _0^I$, with moderate amplitude oscillations for the firing rate of the inhibitory population $R_I$ and low amplitude oscillations for the excitatory population $R_E$. Interestingly, the two oscillatory solutions co-exist for $\eta _0^I\approx -7.5$ to 2.5. Jansen and Rit (Jansen and Rit 1995) demonstrated that transitions between seizures and healthy brain activity could be viewed as transitions between co-existing oscillatory solutions. A similar approach for the next generation neural mass model (without gap junctions) can be found in Byrne et al. (2020). Turning off the gap-junction coupling in the inhibitory population but switching it on for the excitatory population ((c) $\kappa _v^E=0.5$, $\kappa _v^I=0$), does not affect the amplitude of the original branch of oscillatory solutions. There is again an additional branch of oscillatory solutions, this time with very low amplitude $R_I$ oscillations (green/blue line at $R_I\sim 0.7$) and moderate amplitude oscillations in $R_E$ (not shown). With gap junctions switched on in both populations ((d) $\kappa _v^E=\kappa _v^I=0.5$), the 3 oscillatory solution branches exist. For moderate values of $\eta _0^I$, there is a high amplitude oscillation, a moderate amplitude oscillation (low amplitude oscillations in $R_E$) and a low amplitude oscillation (moderate amplitude oscillations in $R_E$).

With a good understanding of the behaviour of the spatially clamped system, we move on to consider the spatially extended neural field model.

## Neural Field Model

Brain waves are inherently dynamical phenomena and come in a vast variety of forms that can be observed with a wide range of neuroimaging modalities. For example, at the mesoscopic scale it is possible to observe a rich repertoire of wave patterns, as seen in voltage-sensitive dye imaging data from the primary visual cortex of the awake monkey (Muller et al. 2014), and local field potential signals across the primary motor cortex of monkeys (Rubino et al. 2006). At the whole brain scale they can manifest as EEG alpha oscillations propagating over the scalp (Hindriks et al. 2014), and as rotating waves (defined as a significant increase in phase offset with rotation about a wave center) seen during human sleep spindles with intracranial electrocorticogram recordings (Muller et al. 2016). Waves are known to subserve important functions, including visual processing (Sato et al. 2012), saccades (Zanos et al. 2015), and movement preparation (Rubino et al. 2006). They can also be associated with dysfunction and in particular epileptic seizures (Martinet et al. 2017). Computational modelling is a very natural way to investigate the mechanisms for their occurrence in brain tissue, as well as how they may evolve and disperse (Heitmann et al. 2013, 2017; Liou et al. 2020).

The study of cortical waves (at the scale of the whole brain) is best advanced using a continuum description of neural tissue. The most common of these are referred to as neural fields, and are natural extensions of neural mass models to incorporate anatomical connectivity and the associated delays that arise through wiring up distant regions using axonal fibres. The study of waves, their initiation, and their interactions is especially pertinent to the study of epileptic brain seizures and it is known that gap junctions are especially important in this context (Martinet et al. 2017). Phenomenological neural field models with gap-junction coupling have previously been developed and analysed by Steyn-Ross et al. (2007, 2012), and more principled ones derived from theta-neuron models by Laing (2014, 2015). In the latter approach it was necessary to overcome a technical difficulty by regularising the shape of the action potential. However, with the approach used in ﻿“Neural mass model” section this is not necessary and the neural field version of (3)–(4) is constructed by replacing full temporal derivatives by partial temporal derivatives and replacing the temporal dynamics for *U* with the dynamics $QU= \Psi$, where $\Psi$ denotes a spatio-temporal drive. For example, in the plane we might consider

6 $$\begin{aligned} \Psi (\mathbf {r},t) = \int _{\mathbb {R}^2} w(|\mathbf {r} -\mathbf {r}'|) R (\mathbf {r}', t - |\mathbf {r} -\mathbf {r}'|/c) \mathrm{d}\mathbf {r} ', \end{aligned}$$

where $R(\mathbf {r},t)$ is the population firing rate at position $\mathbf {r} \in \mathbb {R}^2$ at time *t* and *c* represents the speed of an action potential, as illustrated in Fig. 1. Typical values for cortico-cortical axonal speeds in humans are distributed, and appear to peak in the $5-10$ m/s range (Nunez and Srinivasan 2014). Here, *w* represents structural connectivity as determined by anatomy. For example, long-range corticocortical interactions are predominantly excitatory whilst inhibitory interactions tend to be much more short-ranged, suggesting a natural choice for the shape of *w* as an inverted Mexican hat (Stepanyants et al. 2009). A similar equation would hold in one spatial dimension. We emphasise that in the continuum model presented here, the gap-junction coupling has no spatial extent. The mass model (defined locally) incorporates gap junctions, while the only coupling between masses is via synaptic currents. The model in Laing (2014) treats a linear array with nearest neighbour electrical interactions (representing cells that touch) as well as allowing for interactions beyond nearest neighbour. For large scale brain modelling it is more natural to view the brain as a network of synaptically interacting neural masses, each with its own local synaptic and gap-junction currents, with longer range interactions mediated only by synaptic currents.

In this section we shall work with the explicit choices of structural connectivity $w(x) = (|x|-1)\mathrm{e}^{-|x|}$ in 1D and $w(r) = (r/2-1) \mathrm{e}^{-r}/(2 \pi )$ in 2D (where *x* represents distance in 1D, and *r* represents radial distance in 2D). For convenience we have chosen spatial units so that the scale of exponential delay is unity, though note that typical values for the decay of excitatory connections between cortical areas (at least in macaque monkeys) is $\sim 10$ mm (Markov et al. 2010). Both of the above kernel shapes have an inverted wizard hat shape and are balanced in the sense that the integral over the whole domain is zero. They also allow for a reformulation of the neural field model as a partial differential equation, as detailed in Appendix 3. The resulting brain-wave equation is very amenable to numerical simulation using standard (e.g. finite difference) techniques. Before we do this, it is first informative to determine some of the patterning properties of the neural field model using a Turing instability analysis. Below we outline the results of the analysis and discuss the ensuing patterns for the neural field model in both 1D and 2D.

### One Spatial Dimension

Turing instability analysis, originally proposed by Turing in 1952 (Turing 1952), is a mechanism for exploring the emergence of patterns in spatio-temporal system, including neural fields. Similar to the bifurcation analysis for the neural mass model, it allows us to determine the parameter values for which oscillations and patterns occur. Bulk oscillations, whereby synchronous activity across the spatial domain varies uniformly at the same rate, emerge at a Hopf bifurcation. Static patterns, which do not change with time, emerge at a Turing bifurcation. Dynamic patterns, that oscillate in time and space, emerge at a Turing–Hopf bifurcation.

**Fig. 8:**
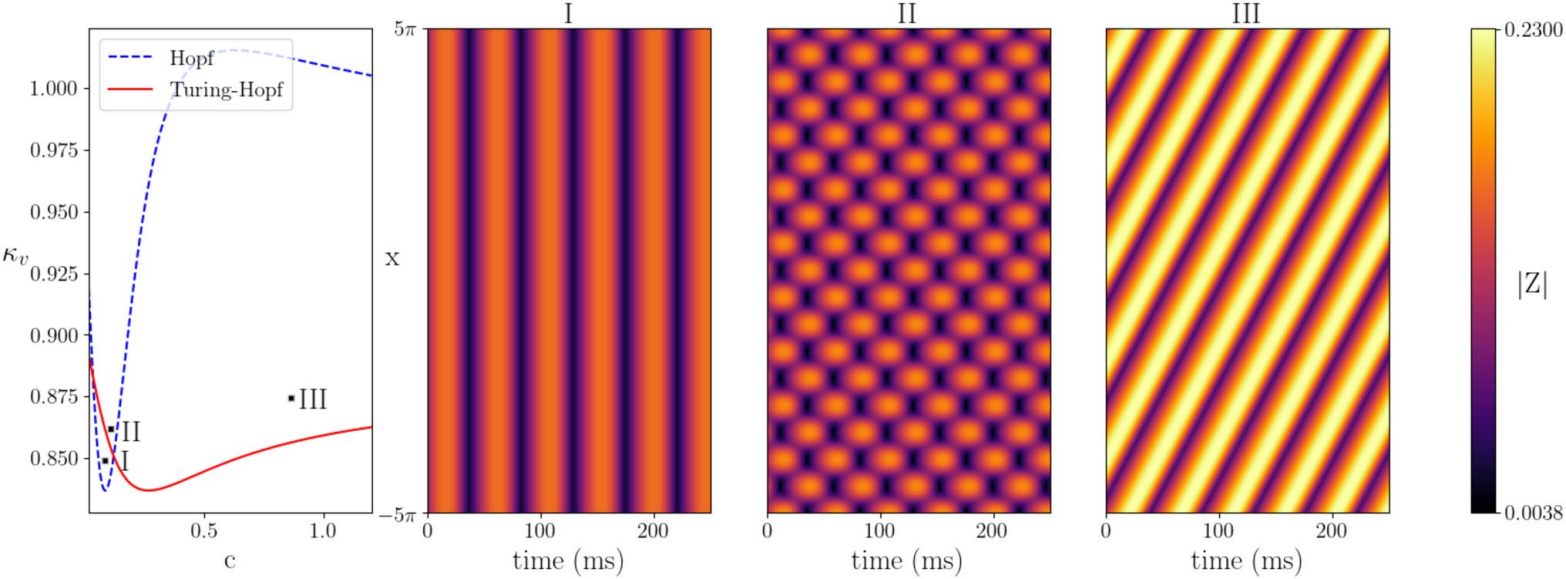
Turing instability analysis for the one-dimensional neural field model. The left panel shows the Hopf and Turing–Hopf curves as a function of the action potential speed *c* and gap-junction coupling strength $\kappa _v$. Above these curves patterned states emerge. The three right hand panels show simulations near Hopf, and two Turing–Hopf points: (I) Bulk oscillation with $c = 0.1$, $\kappa _v = 0.85$, (II) Standing wave with $c = 0.11$, $\kappa _v = 0.855$, (III) Periodic travelling wave with $c = 1.0$, $\kappa _v = 0.88$. Other parameter values: $\eta _0 = 1$, $\kappa _s = 10$, $\tau =15$, $\alpha =0.5$, $\gamma =0.5$

The 1D neural field model, given in Appendix 3 by (38), supports both bulk oscillations and spatio-temporal patterns. Using the inverted wizard hat connectivity kernel (long-range excitation and short-range inhibition), we find Hopf and Turing–Hopf bifurcations (Fig. 8 left). See Appendix 4 for details of the analysis. For the chosen parameter values and weak gap-junction coupling ($\kappa _v \lesssim 0.8$), the spatially-uniform steady state is always stable and neither patterns nor oscillations exist. Increasing the median background drive $\eta _0$ moves the Hopf and Turing–Hopf curves down in the *c*-$\kappa _V$ plane, allowing for oscillations and patterns in the absence of gap junctions ($\kappa _v =0$). For slow action potential speeds ($c\lesssim 0.2$), the system first undergoes a Hopf bifurcation as $\kappa _v$ is increased and bulk oscillations emerge (Fig. 8I). As $\kappa _v$ is increased further, the system undergoes a Turing–Hopf bifurcation and standing waves emerge (Fig. 8II). For faster action potential speeds ($c \gtrsim 0.2$), the Turing–Hopf bifurcation occurs before the Hopf, and we see periodic travelling and standing waves between the two bifurcations (Fig. 8III).

**Fig. 9:**
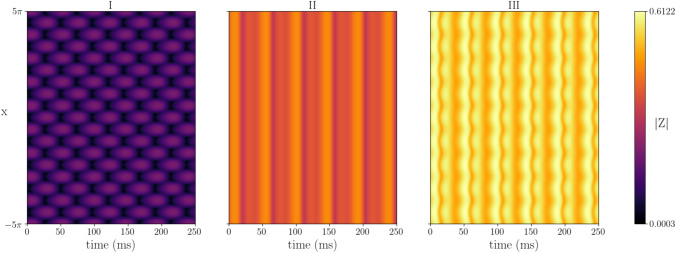
Simulations of the one-dimensional neural field model under variation in $\kappa _v$: (I) Standing wave with $\kappa _v=0.86$, (II) Bulk oscillations with $\kappa _v = 1.0$, (III) Mixed dynamics with $\kappa _v = 1.2$. Other parameters $c = 0.11$, $\eta _0 = 1$, $\kappa _s = 10$, $\tau =15$, $\alpha =0.5$, $\gamma =0.5$

To assess the role of gap junctions, we fixed the action potential speed $c=0.11$ and explored the dynamics of the synchrony variable |*Z*| for different gap-junction coupling strengths $\kappa _v$ (Fig. 9). For weak gap-junction coupling (I), there is a regular standing wave and the level of synchronisation is low. As $\kappa _v$ is increased bulk oscillations emerge and the level of synchronisation increases (II). Increasing $\kappa _v$ further leads to the emergence of mixed dynamics, with both spatial and temporal patterning. The tissue is now highly synchronised, confirming the belief that gap-junction coupling increases the level of synchronisation.

For a standard wizard hat coupling kernel (long-range inhibition and short-range excitation) the neural field model can undergo a Turing bifurcation, as well as Hopf and Turing–Hopf bifurcations (see Supplementary material 1 panel (a)). Changing the sign of the synaptic coupling strength $\kappa _s$ changes the coupling to long-range inhibition and short-range excitation. When Turing and Hopf instabilities occur simultaneously, interesting patterns emerge. In particular, we see stationary bumps where the activity at the centre of the bump oscillates in both space and time (see Supplementary material 1 panel (b)). We will discuss the two dimensional version of such patterns in more detail below.

### Two Spatial Dimensions

A Turing analysis was also performed for the 2D neural field equation, given in Appendix 3 by (37), and a very similar bifurcation structure was found (see Supplementary material 2 panel (a)). As expected, close to the Hopf bifurcation the activity of the tissue oscillates in time, but no spatial pattern emerges (see Supplementary material 3). Near the Turing–Hopf bifurcation we see both planar waves (Supplementary material 4) and radial waves (Supplementary material 5) depending on initial conditions. Close to the intersection of the Turing–Hopf and Hopf bifurcation we see mixed spatio-temporal dynamics (Supplementary material 6). Away from bifurcation, more interesting patterns emerge.

**Fig. 10:**
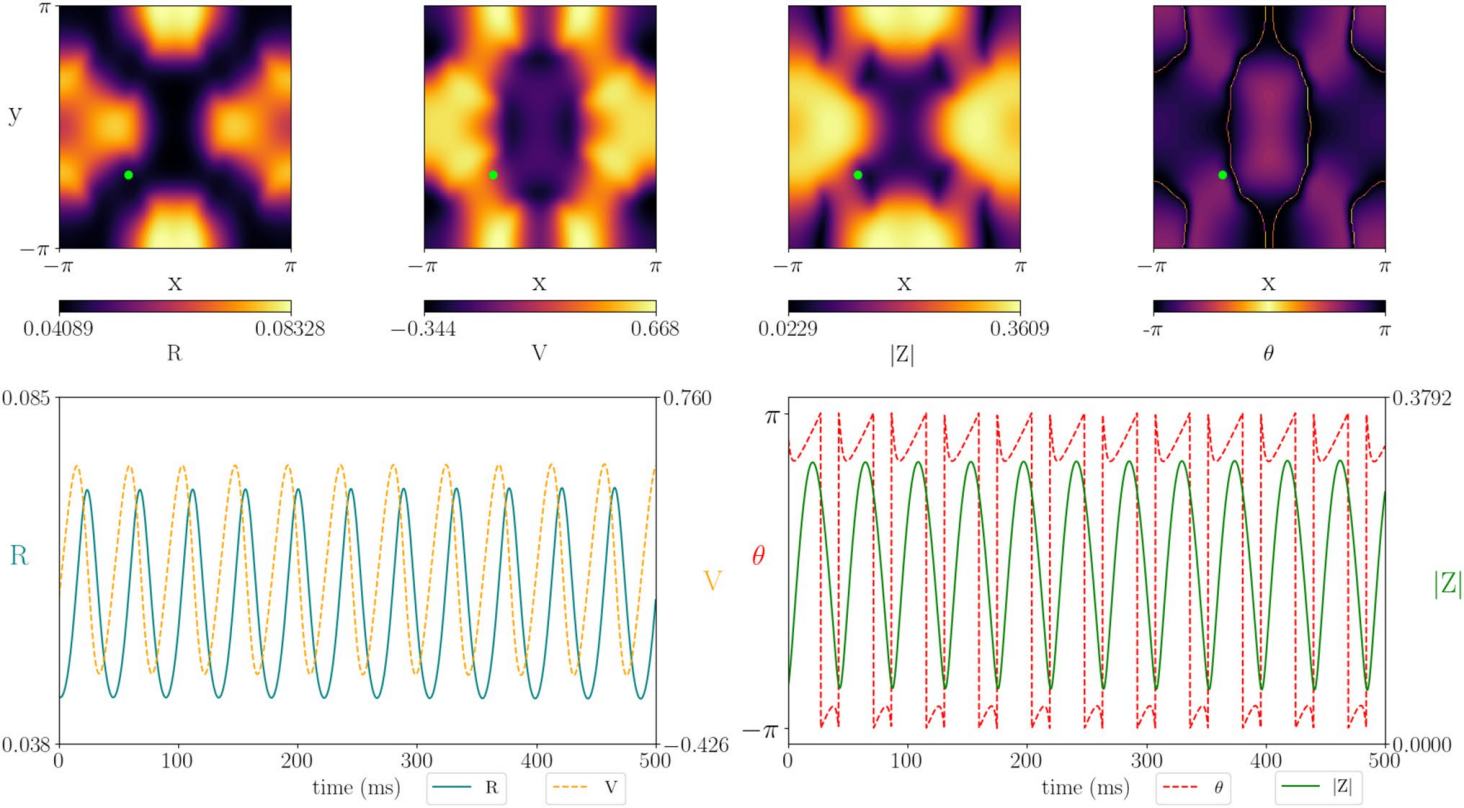
Simulations of the two-dimensional neural field model showing that, beyond a dynamic Turing instability, rotating waves with source and sink dynamics may emerge. Top: a snapshot of a patterned state in the (*R*, *V*) and $(|Z|, \theta )$ variables. Bottom: the corresponding time-series for the point marked by the small green circle in the top panel. A movie illustrating how this pattern evolves in time is given in Supplementary material 7. Parameter values: $c = 1$, $\eta _0 = 2$, $\kappa _v = 0.695$, $\kappa _s = 12$, $\tau =20$, $\alpha =0.5$, $\gamma =0.5$

We fix the action potential speed $c=1$ and vary the gap-junction coupling strength $\kappa _v$ to assess how gap-junction coupling affects patterning. For weak gap-junction coupling, we observe rotating waves with source and sink dynamics where the waves collide with each other (Fig. 10). The domain shown contains 12 rotating cores. Periodic boundary conditions were used. Hence, the cores at the edge of the domain wrap around to those on the other side. Supplementary material 7 shows the temporal evolution of the synchrony variable |*Z*|, from which the cores and rotations are readily observed. The direction of rotation alternates, such that every second core rotates clockwise/anti-clockwise.

**Fig. 11:**
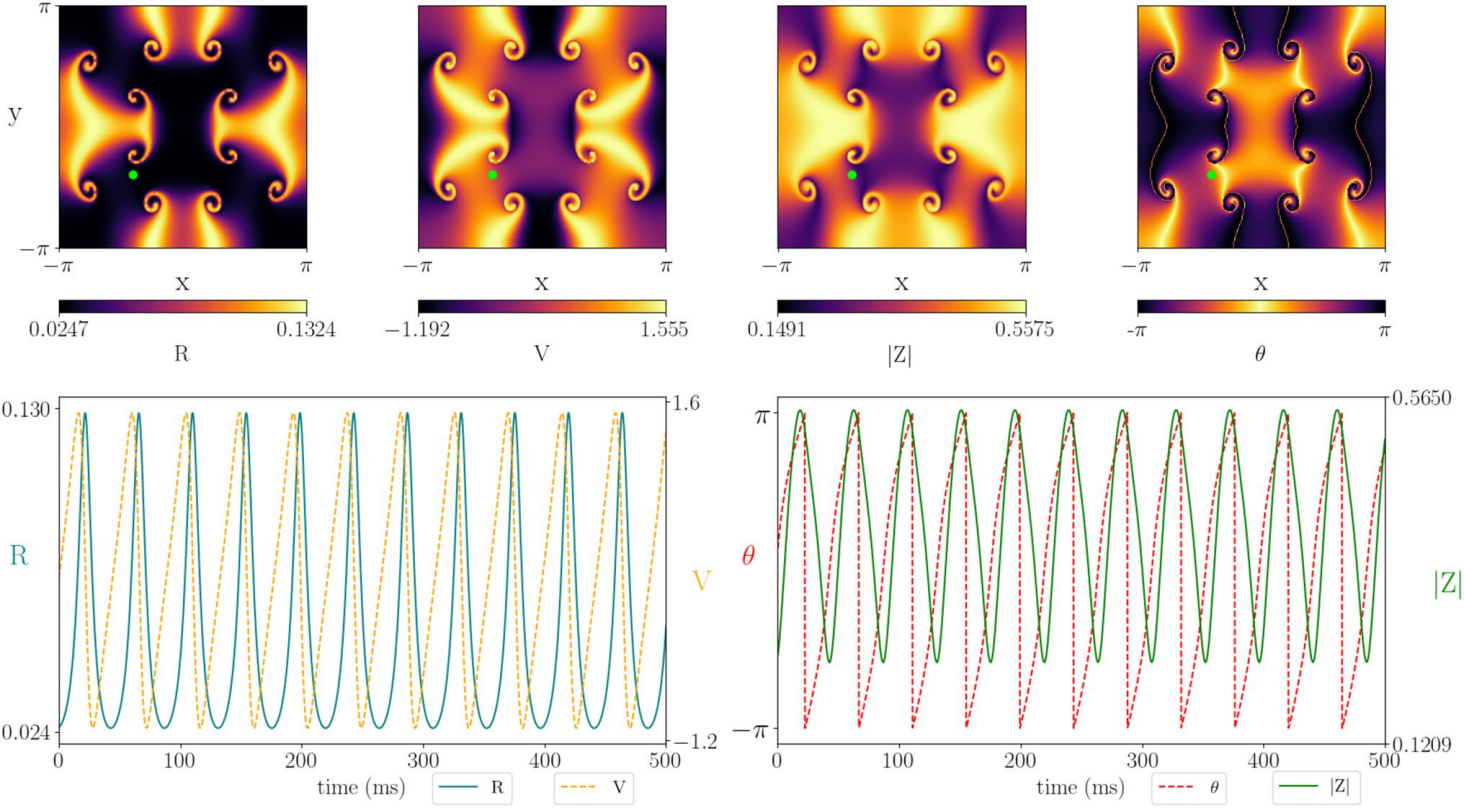
Simulations of the two-dimensional neural field model with moderate gap-junction coupling strength. In this case robust spiral waves emerge at the centre of rotating cores. The spiral is tightly wound with a diffused tail of high amplitude activity that propagates into the rest of the domain and interacts with the other rotating waves. Top: a snapshot of a patterned state in the (*R*, *V*) and $(|Z|, \theta )$ variables. Bottom: the corresponding time-series for the point marked by the small green circle in the top panel. The full spatio-temporal can be seen in Supplementary material 8. Parameter values: $c = 1$, $\eta _0 = 2$, $\kappa _v = 0.8$, $\kappa _s = 12$, $\tau =20$, $\alpha =0.5$, $\gamma =0.5$

As the gap-junction coupling strength $\kappa _v$ is increased robust spirals emerge at the centre of the rotating cores. The spiral is tightly wound with a diffused tail of high amplitude activity that propagates into the rest of the domain and interacts with the other rotating waves (Fig. 11). The time course of a point close to the centre of a rotating core (green dot) depicts higher amplitude oscillations for the firing rate *R*, mean membrane potential *V* and level of synchronisation |*Z*| when compared to the simulations for lower gap-junction coupling strength $\kappa _v$ (Fig. 10). In addition, the peaks in *R* are sharper and the minimum level of synchrony |*Z*| is substantially higher. The temporal evolution for the full tissue can be seen in Supplementary material 8.

We again note that increasing the gap-junction coupling strength increases the level of synchronisation across the tissue. For $\kappa _v=0.695$, the synchrony variable oscillates between 0.02 and 0.36. For $\kappa _v=0.8$, it oscillates between .120 and 0.56. Increasing $\kappa _v$ further does not change the overall dynamics, but does result in higher levels of synchronisation. This supports the hypothesis that gap-junction coupling lends to more synchronous activity.

**Fig. 12:**
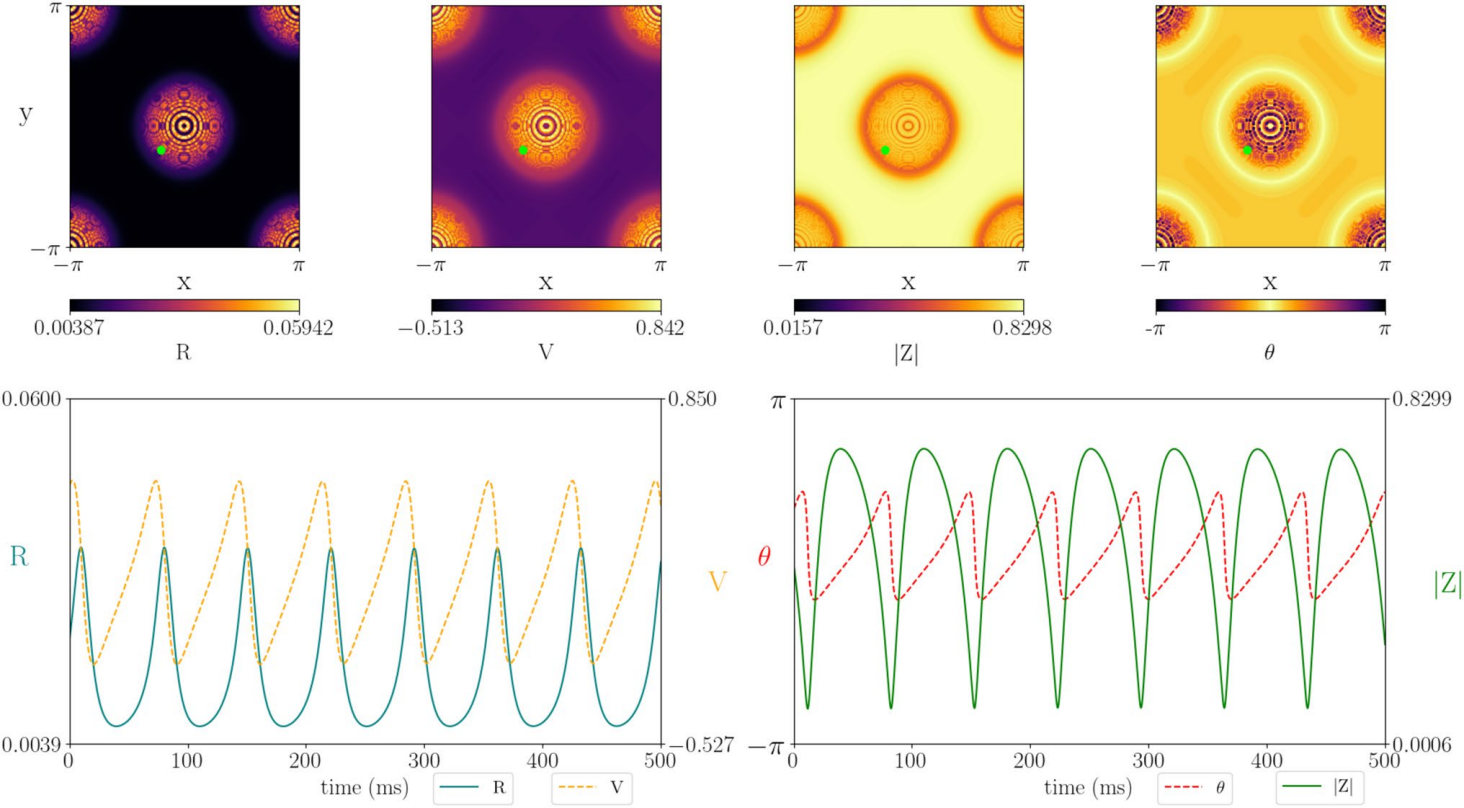
Simulations of the two-dimensional neural field model with short-range excitation and long-range inhibition, showing the emergence of a spatially localised spot solution (top panel). Note that the core of the spot has a rich temporal dynamics, as indicated in the bottom panel showing the time course for a point within the core (green dot in top panel). A movie showing the full spatio-temporal can be found in Supplementary material 9. Parameters values: $c = 10.0$, $\eta _0 = 0.1$, $\kappa _v = 1.0$, $\kappa _s = -25$, $\tau =1$, $\alpha =5$, $\gamma =0.5$

As mentioned in the “One Spatial Dimension” section, for a regular wizard hat connectivity kernel (short-range excitation and long-range inhibition) the neural field supports static Turing patterns, periodic bumps of high activity in 1D and a periodic lattice of high-activity spots in 2D. When a Hopf instability coincides with this Turing instability, patterns form at the centre of these localised states. In 2D, patterns of concentric circles can appear within spots when the two bifurcations coincide (Fig. 12). Activity within a localised state can oscillate in time, while the activity in the surround is constant with a low firing rate. These patterns are reminiscent of chimeras (Abrams and Strogatz 2004; Kuramoto and Battogtokh 2002; Laing 2009a, b; Omelchenko et al. 2011), as seen in networks of coupled oscillators, where a fraction of the oscillators are phase-locked or silent while the others oscillate incoherently. Note how the peaks in firing rate coincide with peaks in synchrony. However, in the surround synchrony is high, but the firing rate is minuscule. This indicates that the neurons are also synchronised at rest. A video illustrating how these exotic patterns evolve on the entire spatial domain is provided in Supplementary material 9 and the bifurcation diagram is given in Supplementary material 2 panel (b). The patterns presented here persist with the refining of the numerical mesh so we are confident that the relatively sharp changes between spatial points are not just a numerical artefact.

When not perfectly synchronised, the relative timing of oscillations across single areas or distant regions in the cortex can give rise to a range of flexible *phase offsets* which can manifest as travelling waves of various shapes, including plane, radial and spiral waves (Muller et al. 2018). The numerical simulations presented above highlight the ease with which these can be generated within the next generation neural field model with local gap-junction currents, and in particular spiral waves. The latter are thought to be highly relevant to *status epilepticus* characterised by the formation of spiral waves that emerge after wavefront annihilation and exhibit complex interactions (Liou et al. 2020).

## Discussion

Mean-field models have proven invaluable in understanding neural dynamics. Although phenomenological in nature, coarse-grained neural mass/field models have proven particularly useful in describing neurophysiological phenomena, such as EEG/MEG rhythms (Zhang 1996), cortical waves (Wilson et al. 2001; Roberts et al. 2019), binocular rivalry (Laing and Chow 2002; Bressloff and Webber 2012), working memory (Laing et al. 2002) and visual hallucinations (Ermentrout and Cowan 1979; Bressloff et al. 2001). The exclusion of synchrony in standard neural mass/field models prohibits them from describing event-related synchronisation and desynchronisation; the increase and decrease of oscillatory EEG/MEG power due to changes in synchrony within the neural tissue. Here we presented and analysed a recently developed neural mass/field model that incorporates within population synchrony. In contrast to other reductive approaches for describing the behaviour of populations of spiking neurons the one described here is *exact* (in the thermodynamic limit) for realistic event-driven models of (non-instantaneous) synaptic process. For example, the spike-density formalism for reducing networks of linear integrate-and-fire neurons requires a moment closure approximation (Ly and Tranchina 2007), whilst Fokker-Planck approaches for describing renewal-type spiking neurons often only reduce after the truncation of some eigenfunction expansion (Pietras et al. 2020).

The mean-field model presented here has previously been applied to real MEG data in Byrne et al. (2017) (at the neural mass level) and used with MRI-derived structural connectivity in Byrne et al. (2020) (for a network of neural masses), though not with the inclusion of gap junctions. The main benefit of such a model is that it is derived from a population of interacting spiking neurons, with the QIF model incorporating a reasonable representation of the action potential shape. This further allows for the inclusion of realistic gap junctions at the cellular level. Gap junctions are known to promote synchrony within neural tissue (Watanabe 1958; Bennett 1977) and the strength of these connections has been linked to the excessive synchronisation driving epileptic seizures (Mylvaganam et al. 2014; Volman et al. 2011). Nonetheless, it is also important to recognise the important effects that the extracellular space has on seizure dynamics, as discussed in Wei et al. (2014). Recent work by Martinet et al. (2017) has emphasised the usefulness of bringing models to bear on this problem, and coupled the Steyn-Ross neural field model (Steyn-Ross et al. 2013) to a simple dynamics for local extracellular potassium concentration. Here, gap junctions are modelled by appending a diffusive term to a standard neural field and increases in the local extracellular potassium concentration act to decrease the inhibitory-to-inhibitory gap-junction diffusion coefficient (to model the closing of gap junctions caused by the slow acidification of the extracellular environment late in seizure). A more refined version of this phenomenological approach would be to replace the Steyn-Ross model with the neural field described here. This would allow a more principled study of how slow changes in the extracellular environment could initiate wave propagation, leading to waves that travel, collide, and annihilate. Indeed, simulations of the next-generation neural field model (without coupling to the extracellular space) have already shown such rich transient dynamics including seizure-like oscillations (and their dependence on the strength of gap-junction coupling). It would be interesting to explore this further, and in particular the transitions whereby spatio-temporal wave patterns are visited in sequence. This has already been the topic of a major modelling study by Roberts et al. (2019) who considered a variety of more traditional neural mass models in a connectome inspired network using the 998-node Hagmann et al. dataset (Hagmann et al. 2008) with a single fixed axonal delay. A similar computational study, with a focus on spiral waves and sinks/sources from which activity emanates/converges, could also be undertaken using the alternative neural mass model presented here, and with the further inclusion of space-dependent axonal delays. Moreover, electrical stimulation can easily be integrated into the model, by returning to the microscopic voltage dynamics model given by (2) (which ensure current balance) and including a time-dependent drive, say *A*(*t*), which could represent a pattern of applied transcranial direct current. This modifies the background drive in the mean-field model according to $\eta _0 \rightarrow \eta _0 + A(t)$. In Byrne et al. (2020) this approach was used to determine the effects of transcranial magnetic stimulation (with an induced electrical form for *A*(*t*)) on patterns of network functional connectivity. Finally, it is well to note the assumption throughout our modelling study that chemical and electrical synapses operate independently. However, there is now accumulating evidence to suggest that this might not be the case (Pereda 2014). For example, neurotransmitter modulators released by nearby synaptic terminals can regulate the synaptic strength of co-localised chemical and electrical synapses through the activation of G protein-coupled metabotropic receptors. All of the above are topics of ongoing investigation and will be reported upon elsewhere.

## Supplementary Information

Below is the link to the electronic supplementary material.

Electronic supplementary material 1 Bumps in the one-dimensional neural field model. (a) Bifurcation diagram for a standard wizard hat coupling kernel. (b) Simulation close to the Turing and Hopf curves $\eta _0=0.02$ and $\kappa _v = 0.3$. Other parameter values: $c = 10$, $\kappa _s = -60$, $\tau =12$, $\alpha =0.5$, $\gamma =0.5$. (PDF 126 kb)

Electronic supplementary material 2 Turing analysis for two-dimensional neural field model. (a) Hopf and Turing-Hopf curves for an inverted wizard hat coupling kernel ($\kappa _s=5$) with $\eta _0=1.2$, $\tau =15$, $\alpha =0.5$, $\gamma =0.5$. (b) Bifurcation diagram for a standard wizard hat coupling kernel ($\kappa _s=-60$), with $c=10$, $\tau =8$, $\alpha =0.5$, $\gamma =0.1$. (PDF 49 kb)

Electronic supplementary material 3 Bulk oscillations appear in the 2D neural field model when we simulate close to the Hopf bifurcation. The entire tissue oscillates coherently and there are no spatial patterns. Here we show the oscillations for the synchrony variable |*Z*|. Parameter values: $\kappa _v = 0.67$, $c = 0.1$, $\eta _0=2$, $\kappa _s = 12$, $\tau =20$, $\alpha =0.5$, $\gamma =0.5$. (M4V 336 kb)

Electronic supplementary material 4 When close to the Turing-Hopf bifurcation, perturbing the 2D neural field model with horizontal bars of high activity results in planar waves. A high activity source forms at the centre of the domain and the waves propagate up and down from it. The evolution of the synchrony variable |*Z*| is shown here. Parameter values: $\kappa _v = 0.693$, $c = 1$, $\eta _0=2$, $\kappa _s = 12$, $\tau =20$, $\alpha =0.5$, $\gamma =0.5$. (M4V 813 kb)

Electronic supplementary material 5 The uniform steady state of the 2D neural field model was perturbed with a Gaussian to initiate radial waves. The temporal evolution of the waves is shown for the synchrony variable |*Z*|. A source of high activity emerges at the centre of the domain and the waves emanate from it. Periodic boundary conditions were used, and as such, the waves interfere when they reach the edge of the domain. Parameter values: $\kappa _v = 0.693$, $c = 1$, $\eta _0=2$, $\kappa _s = 12$, $\tau =20$, $\alpha =0.5$, $\gamma =0.5$. (M4V 1342 kb)

Electronic supplementary material 6 Mixed dynamics of standing waves and bulk oscillations emerge in the 2D neural field model when we are close to the Turing-Hopf and Hopf bifurcation and the uniform steady state is perturbed with horizontal bars of high activity. We show the temporal dynamics of the synchrony variable |*Z*| in this movie. Parameter values: $\kappa _v = 0.69$, $c = 0.1$, $\eta _0=2$, $\kappa _s = 12$, $\tau =20$, $\alpha =0.5$, $\gamma =0.5$. (M4V 936 kb)

Electronic supplementary material 7 Simulation of the 2D neural field model corresponding to the weak gap-junction coupling regime shown in Fig. 10. The temporal evolution of synchrony variable |*Z*| is shown here. The steady state was perturbed with a spatially periodic lattice and rotating waves emerge. We observe source and sink dynamics were the waves interact with one another. Parameter values: $\kappa _v = 0.695$, $c = 1$, $\eta _0 = 2$, $\kappa _s = 12$, $\tau =20$, $\alpha =0.5$, $\gamma =0.5$. (M4V 1855 kb)

Electronic supplementary material 8 Spatio-temporal dynamics of the synchrony variable |*Z*| in the 2D neural field model with moderate gap-junction coupling. The simulation corresponds to Fig. 11, where tightly wound spirals appear the centre of the rotating waves. Parameter values: $c = 2$, $\eta _0 = 6$, $\kappa _v = 0.6$, $\kappa _s = 12$, $\tau =1$, $\alpha =5$, $\gamma =0.5$. (M4V 2888 kb)

Electronic supplementary material 9 Simulation of the chimera dynamics in the 2D neural field model with a regular wizard hat coupling kernel. Movie corresponds to Fig. 12 and shows the dynamics of the synchrony variable |*Z*|. Parameters values: $c = 10.0$, $\eta _0 = 0.02$, $\kappa _v = 0.6$, $\kappa _s = -60$, $\tau =8$, $\alpha =0.5$, $\gamma =0.5$. (M4V 2237 kb)

## Data Availability

Code for running the 1D and 2D neural field simulations can be found at https://github.com/Jamesafross/Neural_Field_with_gaps.

## Appendix 1: Mean-Field Reduction

Consider a heterogeneous network of *N* quadratic integrate-and-fire neurons with voltage $v_i$ and both gap-junction and synaptic coupling:

7 $$\begin{aligned} \tau \dot{v}_i = \eta _i+v_i^2 + \frac{\kappa _v}{N} \sum _{j=1}^N (v_j - v_i) + \frac{\kappa _s}{N} \sum _{j=1}^N \sum _{m \in \mathbb {Z}} s(t- T_j^m), \end{aligned}$$

$i=1,\ldots , N$, with $v_\text {r} \le v_i \le v_\text {th}$. Here, the *m*th firing time of the *j*th neuron is defined implicitly by $v_j(T_j^m)=v_\text {th}$. The network nodes are subject to reset: $v_i \rightarrow v_\text {r}$ at times $T_i^m$. The strengths of gap-junction and synaptic coupling are $\kappa _v$ and $\kappa _s$ respectively. The function *s*(*t*) represents the shape of a post synaptic response (to a delta-Dirac spike) and will be taken to be the Green’s function of a linear differential operator *Q*. For an alpha-function $s(t) = \alpha ^2 t \exp (-\alpha t) H(t)$, where *H* is a Heaviside function, $Q=(1+\alpha ^{-1} \mathrm{d}/\mathrm{d}t)^2$, whilst for an exponential response $s(t) = \alpha \exp (-\alpha t) H(t)$, $Q=(1+\alpha ^{-1} \mathrm{d}/\mathrm{d}t)$. In (7) the $\eta _i$ are random variables drawn from a Lorentzian distribution:

8 $$\begin{aligned} g (\eta ) = \frac{1}{\pi } \frac{\gamma }{(\eta -\eta _0)^2+ \gamma ^2}, \end{aligned}$$

with median $\eta _0$ and half-width $\gamma$. The threshold $v_\text {th}$ and reset $v_\text {r}$ values are taken to be $\infty$ and $-\infty$, respectively.

To derive the mean-field equations we follow closely the exposition by Montbrió et al. (2015). Consider the thermodynamic limit $N \rightarrow \infty$ with a distribution of voltage values $\rho (\upsilon |\eta ,t)$. The continuity equation for $\rho$ is

9 $$\begin{aligned} \frac{{\partial \rho }}{{\partial t}} + \frac{{\partial (\rho \dot{\upsilon })}}{{\partial \upsilon }} = 0, \end{aligned}$$

where

10 $$\begin{aligned} \tau \dot{v}&= \eta +v^2 - \kappa _v v + \kappa _v V+ \kappa _s U, \end{aligned}$$

11 $$\begin{aligned} Q U&= R, \end{aligned}$$

and

12 $$\begin{aligned} V(t)&= \lim _{N \rightarrow \infty } \frac{1}{N}\sum _{j=1}^N v_j, \end{aligned}$$

13 $$\begin{aligned} R(t)&= \lim _{N \rightarrow \infty } \frac{1}{N} \sum _{j=1}^N \sum _{m \in \mathbb {Z}} \delta (t- T_j^m) , \end{aligned}$$

which represent the average voltage and population firing rate respectively. We now assume a solution $\rho (v | \eta , t)$ of the form

14 $$\begin{aligned} \rho (v | \eta , t) = \frac{1}{\pi } \frac{x (\eta ,t)}{(v-y(\eta ,t))^2+x^2(\eta , t)} . \end{aligned}$$

For a fixed $\eta$ the firing rate $r(\eta ,t)$ can be calculated as $\rho (v \rightarrow \infty | \eta , t) \dot{v}(v \rightarrow \infty | \eta , t)$, from which we may establish that

15 $$\begin{aligned} x(\eta ,t) = \pi \tau r (\eta ,t). \end{aligned}$$

By exploiting the structure of (14), with poles at $v_\pm = y \pm i x$, a contour integration shows that

16 $$\begin{aligned} y(\eta ,t) = \text {PV} \, \int _{-\infty }^ \infty \, v \rho (v | \eta , t) \, \mathrm{d}v , \end{aligned}$$

where $\text {PV}$ denotes the Cauchy principal value. After averaging over the distribution of single neuron drives given by (8) we obtain

17 $$\begin{aligned} R(t)&= \frac{1}{\pi \tau } \int _{-\infty }^\infty \mathrm{d}\eta \, x (\eta , t) g(\eta ) , \end{aligned}$$

18 $$\begin{aligned} V(t)&=\int _{-\infty }^\infty \mathrm{d}\eta \, y(\eta , t) g(\eta ) . \end{aligned}$$

For fixed $\eta$, substitution of (14) into the continuity equation and balancing powers of *v* shows that *x* and *y* obey two coupled differential equations that can be written as

19 $$\begin{aligned} \tau \frac{\partial \omega }{\partial t} = - \kappa _v \omega +i \left[ \eta +\kappa _v V + \kappa _s U -\omega ^2 \right] , \end{aligned}$$

where $\omega (\eta ,t) = x(\eta ,t)+iy(\eta ,t)$. After evaluating the integrals in (17) and (18) using contour integration (and using the fact that $\rho$ has poles at $\eta _\pm = \eta _0\pm i \gamma$) the coupled equations for (*R*, *V*) can be found as

20 $$\begin{aligned} \tau \dot{R}&= - \kappa _v R + 2 R V + \frac{\gamma }{\pi \tau } , \end{aligned}$$

21 $$\begin{aligned} \tau \dot{V}&= \eta _0 + V^2- \pi ^2 \tau ^2 R^2 + \kappa _s U. \end{aligned}$$

The complex quantity $W=\pi \tau R +i V$ is known to be related to the Kuramoto order parameter *Z* by the conformal map (Montbrió et al. 2015):

22 $$\begin{aligned} Z = \frac{1-W^*}{1+W^*} , \end{aligned}$$

where $W^*$ is the complex conjugate of *W*. The evolution equation for *Z* is given by the complex differential equation

23 $$\begin{aligned} \tau \dot{Z}&= \frac{\kappa _v}{2} (1-Z^2) -\frac{\gamma }{2} (1+Z)^2 - \frac{i}{2} (1-Z)^2 \nonumber \\&\quad + \frac{i}{2} (1+Z)^2 \left[ \eta _0 + \kappa _v V(Z) + \kappa _s U \right] , \end{aligned}$$

where $$Q U = R(Z)$$ and

24 $$\begin{aligned} R(Z)&= \frac{1}{\pi \tau } \text {Re} \, \left( \frac{1-Z^*}{1+Z^*} \right) , \end{aligned}$$

25 $$\begin{aligned} V(Z)&= \text {Im} \, \left( \frac{1-Z^*}{1+Z^*} \right) . \end{aligned}$$

## Appendix 2: Interacting Sub-populations

Consider an excitatory population labelled by *E* coupled to an inhibitory one labelled by *I*. In this case there are four distinct synaptic inputs with connection strengths $\kappa _s^{ab}$, $a,b \in \{E,I\}$, with $\kappa _s^{aE}>0$ and $\kappa _s^{aI}<0$. Each population has a background drive drawn from a Lorentzian with median $\eta _0^a$ and half-width $\gamma _a$, $a \in \{E,I\}$. Generalising the mean-field model derived in section Appendix 1, for gap-junction coupling only within a given sub-population, we have that

26 $$\begin{aligned} \tau _a \dot{R}_a&= - \kappa _v^a R_a + 2 R_a V_a + \frac{\gamma _a}{\pi \tau _a} , \end{aligned}$$

27 $$\begin{aligned} \tau _a \dot{V}_a&= \eta _0^a + V_a^2- \pi ^2 \tau _a^2 R_a^2 + \sum _{b \in \{ E, I\}} \kappa _s^{ab} U_{ab}, \end{aligned}$$

28 $$\begin{aligned} Q_{ab} U_{ab}&= R_b, \qquad a,b \in \{E,I\}. \end{aligned}$$

For a second order synapse with time-scale $\alpha _{ab}^{-1}$ we would set

29 $$\begin{aligned} Q_{ab} = \left( 1 + \frac{1}{\alpha _{ab}} \frac{\mathrm{d}}{\mathrm{d}t} \right) ^2 . \end{aligned}$$

Note that in a slightly more general setting that would allow for electrical connections between excitatory and inhibitory sub-populations then we would replace (26) and (27) by

30 $$\begin{aligned} \tau _a \dot{R}_a&= - R_a \sum _{b \in \{ E, I\}} \kappa _v^{ab} + 2 R_a V_a + \frac{\gamma _a}{\pi \tau _a} , \end{aligned}$$

31 $$\begin{aligned} \tau _a \dot{V}_a&= \eta _0^a + V_a^2- \pi ^2 \tau _a^2 R_a^2 + \sum _{b \in \{ E, I\}} \kappa _s^{ab} U_{ab} \nonumber \\&\quad+ \sum _{b \in \{ E, I\}} \kappa _v^{ab} [V_b-V_a], \end{aligned}$$

where there are three distinct gap-junctions strengths $\kappa _v^{ab}$, $a,b \in \{E,I\}$, with $\kappa _v^{ab}>0$ and $\kappa _v^{EI}=\kappa _v^{IE}$.

## Appendix 3: Brain Wave Equation

A simple continuum model for an effective single population dynamics can be written in the form

32 $$\begin{aligned} \tau \frac{\partial R}{\partial t}&= - \kappa _v R + 2 R V + \frac{\gamma }{\pi \tau } , \end{aligned}$$

33 $$\begin{aligned} \tau \frac{\partial V}{\partial t}&= \eta _0 + V^2- \pi ^2 \tau ^2 R^2 + \kappa _s U \end{aligned}$$

34 $$\begin{aligned} QU&= \Psi , \end{aligned}$$

where $\Psi = w \otimes R$. The symbol $\otimes$ is used to describe spatial interaction within the neural field model, while *w* represents structural connectivity. For example, in the plane we might consider $(R,V,U)=(R(\mathbf {r},t),V(\mathbf {r},t),U(\mathbf {r},t))$, with $\mathbf {r} \in \mathbb {R}^2$ and $t \ge 0$ with

35 $$\begin{aligned}&\left[ w \otimes R\right] (\mathbf {r},t) = \nonumber \\&\int _{\mathbb {R}^2} w(|\mathbf {r} -\mathbf {r}'|) R (\mathbf {r}', t - |\mathbf {r} -\mathbf {r}'|/c) \mathrm{d}\mathbf {r} ', \end{aligned}$$

where *c* represents the speed of an action potential. We note that (35) can be written as a convolution:

36 $$\begin{aligned} \Psi (\mathbf {r},t) = \int _{\mathbb {R}} \mathrm{d}t' \int _{\mathbb {R}^2} \mathrm{d}\mathbf {r}' G(\mathbf {r} - \mathbf {r}', t-t') R (\mathbf {r}', t') , \end{aligned}$$

where $G(r,t) = w(r)\delta (t-r/c)$. For certain choices of *w* it is possible to exploit this convolution structure to obtain a PDE model, often referred to as a *brain-wave equation* (Nunez 1974; Jirsa and Haken 1997).

For the choice of an inverted balanced wizard hat function with $w(r) = (r/2-1) \mathrm{e}^{-r}/(2 \pi )$ this approach yields the following brain-wave equation:

37 $$\begin{aligned}\left[ \left( 1 + \frac{1}{c} \frac{\partial }{\partial t} \right) ^2 - \frac{3}{2} \nabla ^2 \right] ^2 \Psi = - \left\{ \frac{1}{c} \frac{\partial }{\partial t} \left( 1+\frac{1}{c} \frac{\partial }{\partial t} \right) - \frac{3}{2} \nabla ^2 \right\} R . \end{aligned}$$

Note that (37) is only strictly valid for describing long-wavelength solutions. In one spatial dimension and using $w(x) = (|x|-1)\mathrm{e}^{-|x|}$ the brain-wave PDE is

38 $$\begin{aligned}\left[ \left( 1 + \frac{1}{c} \frac{\partial }{\partial t} \right) ^2 - \frac{\partial ^2}{\partial x^2}\right] ^2 \Psi = - 2\left\{ \frac{1}{c} \frac{\partial }{\partial t} \left( 1+\frac{1}{c} \frac{\partial }{\partial t} \right) ^2 - \frac{\partial ^2}{\partial x^2} \left( 2 + \frac{1}{c} \frac{\partial }{\partial t} \right) \right\} R, \end{aligned}$$

and is an exact reduction of $\Psi = w \otimes R$ (Venkov et al. 2007).

## Appendix 4: Turing Instability Analysis

Consider the homogeneous steady state of (37) given by $(U(\mathbf {r}, t),\Psi (\mathbf {r}, t),R(\mathbf {r}, t),V(\mathbf {r}, t)) = (0,0,R_0,V_0)$ where $(R_0,V_0)$ are given by the simultaneous solution of the algebraic equations

39 $$\begin{aligned} 0&= -\kappa _v R_0 + 2 R_0 V_0 + \frac{\gamma }{ \pi \tau } , \end{aligned}$$

40 $$\begin{aligned} 0&= \eta _0 +V_0^2 - \pi ^2 \tau ^2 R_0^2 . \end{aligned}$$

We linearise around the steady state and consider perturbations of the form $(U(\mathbf {r}, t),\Psi (\mathbf {r}, t),R(\mathbf {r}, t),V(\mathbf {r}, t)) = (0,0,R_0,V_0) + \epsilon (\overline{U},\overline{\Psi },\overline{R},\overline{V}) \mathrm{e}^{\lambda t} \mathrm{e}^{i \mathbf {k} \cdot \mathbf {r}}$ for $|\epsilon | \ll 1$ and $\lambda =\mu +i\omega$. Substitution into (37) and working to first order in $\epsilon$ gives the linear relationship

41 $$\begin{aligned} \left[ \left( 1 + \frac{\lambda }{c} \right) ^2 + \frac{3}{2} k^2 \right] ^2 \overline{\Psi } = - \left\{ \frac{\lambda }{c} \left( 1+\frac{\lambda }{c} \right) + \frac{3}{2} k^2 \right\} \overline{R} , \end{aligned}$$

where $k=|\mathbf {k}|$. A linearisation for the dynamics of (*R*, *V*) gives

42 $$\begin{aligned} A(\lambda ) \begin{bmatrix} \overline{R} \\ \overline{V} \end{bmatrix} = \begin{bmatrix} 0 \\ \kappa _s \overline{U} \end{bmatrix}, \quad A(\lambda ) = \tau \lambda I_2 - J, \end{aligned}$$

where $I_2$ is the $2 \times 2$ identity matrix and *J* is the Jacobian

43 $$\begin{aligned} J = \begin{bmatrix} - \kappa _v + 2 V_0 &{} 2 R_0 \\ - 2 \pi ^2 \tau ^2 R_0 &{} 2 V_0 \end{bmatrix} . \end{aligned}$$

We may solve (42) using Cramer’s rule to yield

44 $$\begin{aligned} \overline{R} = \frac{1}{|A(\lambda )|} \begin{vmatrix} 0&-2 R_0 \\ \kappa _s\overline{U}&\tau \lambda - 2 V_0 \end{vmatrix} =\frac{2 \kappa _s R_0}{|A(\lambda )| (1+\lambda /\alpha )^2} \overline{\Psi } , \end{aligned}$$

where we have used the fact that $(1+\lambda /\alpha )^2 \overline{U} = \overline{\Psi }$ (from (34)). Substitution of (44) into (41) and demanding a non-trivial solution for $\overline{\Psi }$ leads to the condition $\mathcal {E}(\lambda ,k)=0$, where

45 $$\begin{aligned} \mathcal {E}(\lambda ,k)&= |A(\lambda )| \left( 1 + \frac{\lambda }{\alpha } \right) ^2 \left[ \left( 1 + \frac{\lambda }{c} \right) ^2 + \frac{3}{2} k^2 \right] ^2 \nonumber \\&\quad +2 \kappa _s R_0 \left[ \frac{\lambda }{c} \left( 1+\frac{\lambda }{c} \right) + \frac{3}{2} k^2 \right] . \end{aligned}$$

Thus, the continuous spectrum $\lambda =\lambda (k)$ is given by the roots of an eight order polynomial.

A similar analysis of (38) gives

46 $$\begin{aligned} \mathcal {E}(\lambda ,k)&= |A(\lambda )| \left( 1 + \frac{\lambda }{\alpha } \right) ^2 \left[ \left( 1 + \frac{\lambda }{c} \right) ^2 + k^2 \right] ^2 \nonumber \\&\quad +4 \kappa _s R_0 \left[ \frac{\lambda }{c} \left( 1+\frac{\lambda }{c} \right) ^2 + k^2 \left( 2 + \frac{\lambda }{c} \right) \right] . \end{aligned}$$

The system undergoes a bifurcation when a branch of solutions $\lambda (k)$ to $\mathcal {E}(\lambda ,k)=0$ touches the imaginary axis, $\mu (k_c) =0$, where $k_c$ is the critical wave number. By the implicit function theorem, this occurs when

47 $$\begin{aligned} \frac{\partial \mathcal {M}}{\partial k}\frac{\partial \mathcal {N}}{\partial \omega }-\frac{\partial \mathcal {M}}{\partial \omega }\frac{\partial \mathcal {N}}{\partial k}=0, \end{aligned}$$

where $$\mathcal {M}=\text {Re} \, ({\mathcal {E}})$$ and $$\mathcal {N}=\text {Im} \, ({\mathcal {E}})$$.

A Hopf bifurcation of the spatially uniform state can be found by solving $\mathcal {E}(i\omega ,0)=0$ for $\omega$. A static Turing bifurcation is found by solving $\mathcal {E}(0,k_c)=0$ and (47) for $k_c$ non-zero, while a dynamic Turing-Hopf bifurcation is found by solving $\mathcal {E}(i\omega ,k_c)=0$ and (47) for non-zero $\omega$ and $k_c$. Interesting patterns tend to emerge when a Hopf and Turing-Hopf intersect at a codimension-2 point. Such a bifurcation can be found by solving $\mathcal {E}(i\omega _1,0)=0$, $\mathcal {E}(i\omega _2,k_c)=0$ and (47) simultaneously for $\omega _1$, $\omega _2$ and $k_c$.

## Appendix 5: Computation and Simulation of the Model

All bifurcations diagrams were generated using XPPAUT (Ermentrout 2002). The neural mass and neural field models were numerically solved using a finite difference scheme on Julia (Bezanson et al. 2017) using a default adaptive solver from the DifferentialEquations.jl package (Rackauckas and Nie 2017).
