# Supplementary figures and images for: Mean-Field Models for EEG/MEG: From Oscillations to Waves
Source: Brain Topogr. 2021 May 15;35(1):36–53. doi: 10.1007/s10548-021-00842-4 (PMC8813727; doi:10.1007/s10548-021-00842-4)

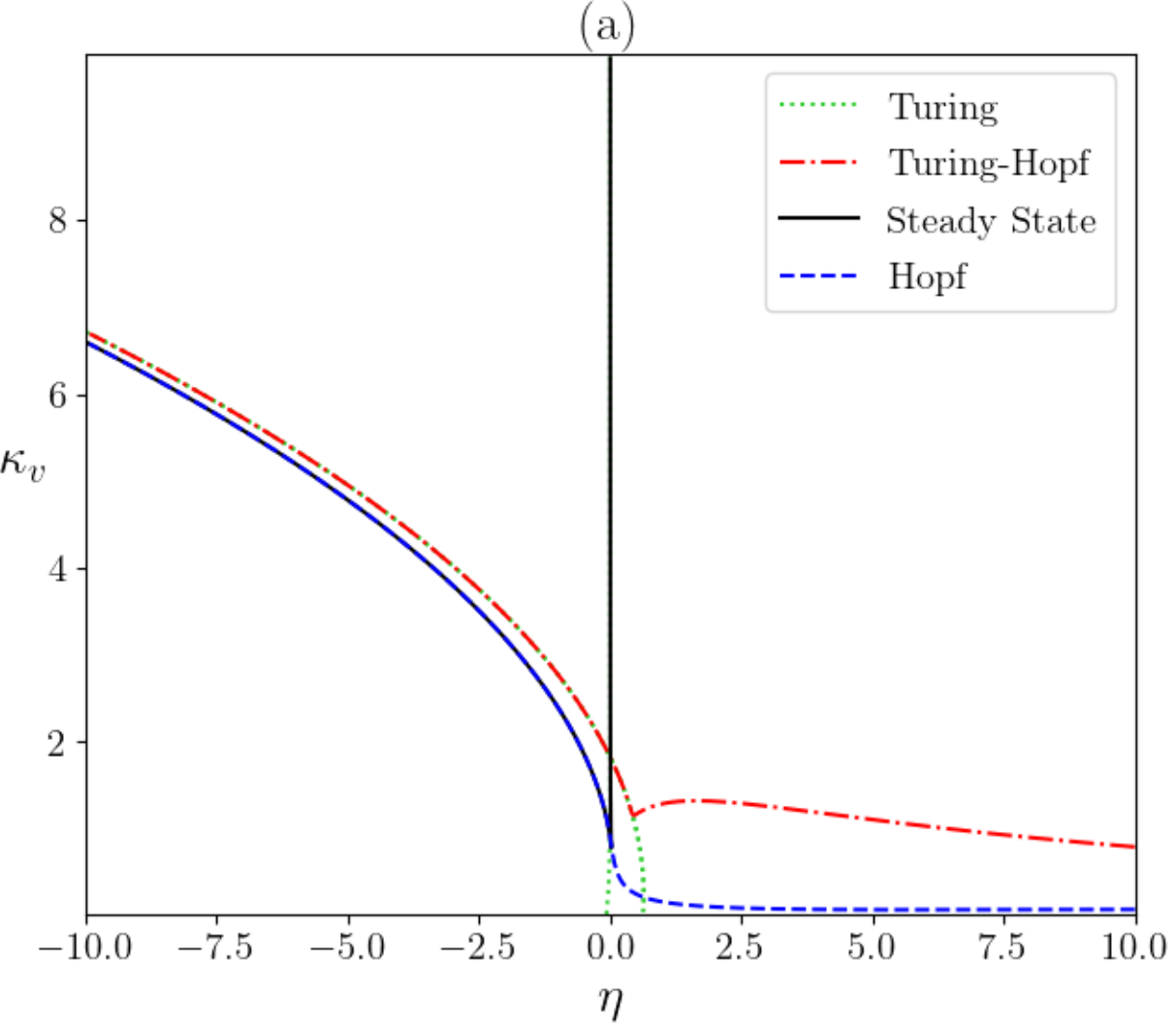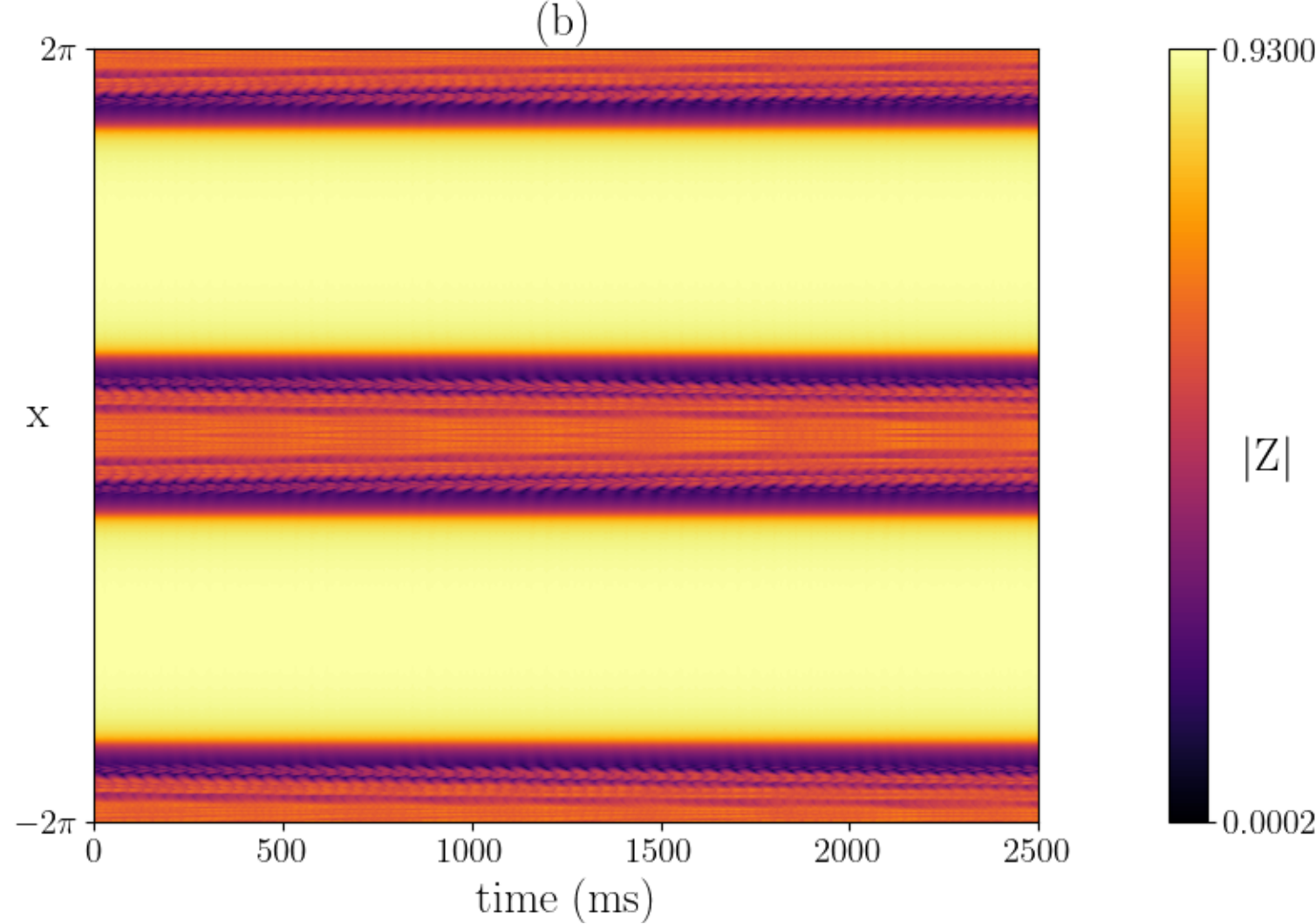

Supplement: Supplementary file 1 — Electronic supplementary material 1 Bumps in the one-dimensional neural field model. (a) Bifurcation diagram for a standard wizard hat coupling kernel. (b) Simulation close to the Turing and Hopf curves $\eta _0=0.02$η0=0.02 and $\kappa _v = 0.3$κv=0.3. Other parameter values: $c = 10$c=10, $\kappa _s = -60$κs=-60, $\tau =12$τ=12, $\alpha =0.5$α=0.5, [file 10548_2021_842_MOESM1_ESM.pdf]

(a)

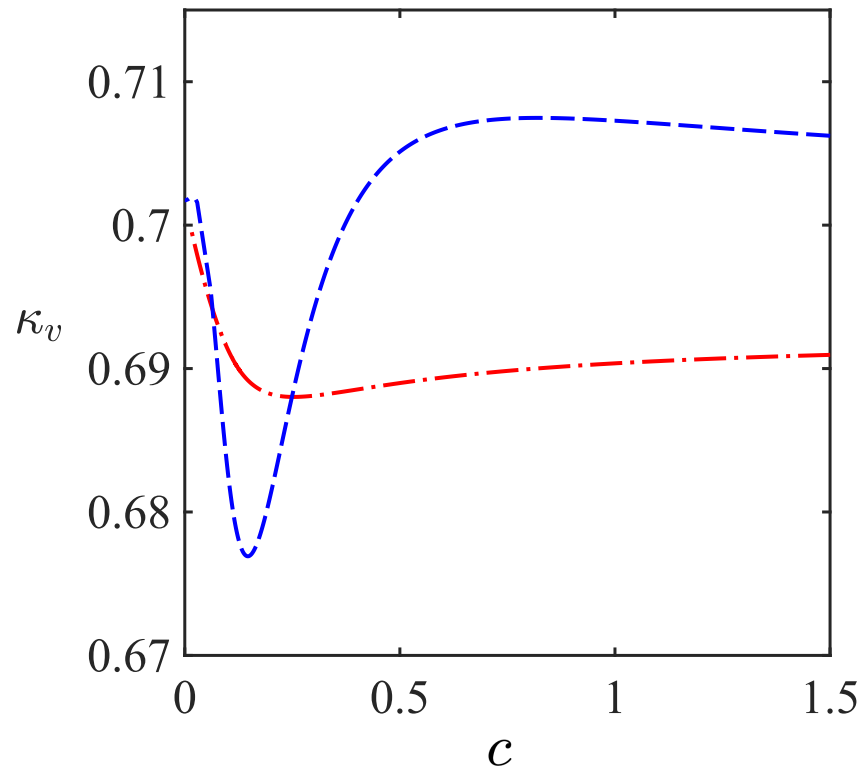

(b)

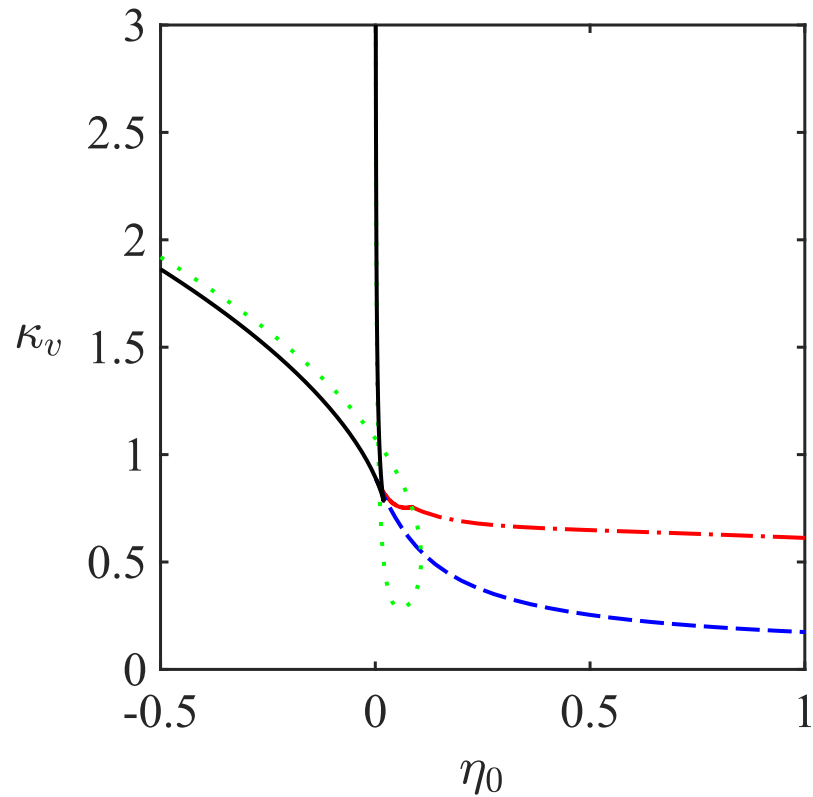

Supplement: Supplementary file 2 — Electronic supplementary material 2 Turing analysis for two-dimensional neural field model. (a) Hopf and Turing-Hopf curves for an inverted wizard hat coupling kernel ($\kappa _s=5$κs=5) with $\eta _0=1.2$η0=1.2, $\tau =15$τ=15, $\alpha =0.5$α=0.5, $\gamma =0.5$γ=0.5. (b) Bifurcation diagram for a standard wizard hat coupling kernel ($\kappa _s=-60$κs=-60), with [file 10548_2021_842_MOESM2_ESM.pdf]
